# Updates on Responsive Drug Delivery Based on Liposome Vehicles for Cancer Treatment

**DOI:** 10.3390/pharmaceutics14102195

**Published:** 2022-10-15

**Authors:** Maria P. Nikolova, Enamala Manoj Kumar, Murthy S. Chavali

**Affiliations:** 1 Department of Material Science and Technology, University of Ruse “A. Kanchev”, 8 Studentska Str., 7017 Ruse, Bulgaria; 2 Bioserve Biotechnologies (India) Private Limited, Unit: D4-7, 1st Floor, Industrial Estate, Moula-Ali, Hyderabad 500040, Telangana, India; 3 Office of the Dean (Research), Division of Chemistry, Department of Science, Faculty of Science & Technology, Alliance University (Central Campus), Chandapura-Anekal Main Road, Bangalore 562106, Karnataka, India

**Keywords:** liposomes, drug delivery, cancer, smart stimulus-responsive, internal and external stimuli

## Abstract

Liposomes are well-known nanoparticles with a non-toxic nature and the ability to incorporate both hydrophilic and hydrophobic drugs simultaneously. As modern drug delivery formulations are produced by emerging technologies, numerous advantages of liposomal drug delivery systems over conventional liposomes or free drug treatment of cancer have been reported. Recently, liposome nanocarriers have exhibited high drug loading capacity, drug protection, improved bioavailability, enhanced intercellular delivery, and better therapeutic effect because of resounding success in targeting delivery. The site targeting of smart responsive liposomes, achieved through changes in their physicochemical and morphological properties, allows for the controlled release of active compounds under certain endogenous or exogenous stimuli. In that way, the multifunctional and stimuli-responsive nanocarriers for the drug delivery of cancer therapeutics enhance the efficacy of treatment prevention and fighting over metastases, while limiting the systemic side effects on healthy tissues and organs. Since liposomes constitute promising nanocarriers for site-targeted and controlled anticancer drug release, this review focuses on the recent progress of smart liposome achievements for anticancer drug delivery applications.

## 1. Introduction

Cancer is thought to be a health problem with the leading cause of death [1]. The number of cancer cases is estimated to reach 21 million by 2030 [2]. Conventional chemo, radiotherapy, and hormone therapy are considered to be ineffective, due to low toxicity, non-specific distribution, and adverse effects [3]. Additionally, most conventional drugs suffer from poor pharmacokinetics, high toxicity, and reduced bioavailability. The field of nanotechnology has surged to a new height, which has inspired many researchers to produce a safer and more efficient drug delivery system using nanotechnology in the treatment of cancer therapy. The treatment of cancer with nanotechnology (nanooncology) has improved treatment efficacy by penetrating deep inside the body, where even the drug cannot reach [4]. The nanotechnologies hold numerous advantages in drug delivery systems, and a few of them include improving the in vivo pharmacokinetic process, enhancing the stability and longevity of the drug in blood circulation, and even modifying the carriers by targeting ligands on their surface for tissue or cell-specific delivery [5].

Significant achievements have occurred in the last few decades by applying injectable drug delivery systems (DDS) for cancer treatment. These advancements include the application of different nanoparticles, including liposomes, that conjugate various macromolecules. The nanocarriers, in the form of liposomes, polymeric nanoparticles, and even inorganic nanoparticles, can reach the interior of the cellular/molecular level and can detect the level of disease spread inside our body. Among the several nanoscale drug carriers, liposomes have demonstrated the greatest potential in various clinical applications [6]. Because of their similarities to biological membranes, liposomes offer excellent opportunities for the drug delivery of molecules into the target cells or subcellular compartments [7]. Lipid-based delivery systems offer cytoplasmic delivery by exploiting natural bio-functions, such as membrane fusion [8], because phospholipids are the main components of the biological cell membrane. Thanks to liposomes, it has become possible to increase the pharmacokinetics parameters of drugs, such as circulation time, controlled release, increased solubility, stability, and intercellular concentration [9].

Target-specific nanocarriers for drug delivery enhance the therapeutic efficacy of the loaded moiety by precisely targeting cancerous cells or tissues and preventing the drug from undergoing hepatic metabolism. To attain the desired pharmacotherapy, the nanovesicles should selectively release the drug at the targeted sites with minimum adverse effects. The cancer tissue targeting strategy can be both passive and active. Passive targeting mainly focuses on the pathological conditions of the disease, such as the difference in pore sizes among endothelial cells of cancer microvasculature that are larger than that of the structures of normal capillaries (an effect known as enhanced permeation and retention (EPR)). Only liposomes with diameters varying between 50 and 150 nm were able to avoid phagocytosis, enter blood vessels in the tumor microenvironment [10,11], and escape from capillaries that perfuse tissues, such as the heart, kidneys, and lungs. However, many studies with passive targeted liposomes reported low selectivity that resulted in higher drug accumulation in healthy tissues and organs and low concentration of liposomes within tumor tissues, resulting in treatment failure. Furthermore, cationic charges were also reported to cause lower tumor penetration and non-specific accumulation, while neutral liposomes were able to penetrate deeper into tissue at the expense of a lower cellular uptake [12].

**Figure 1:**
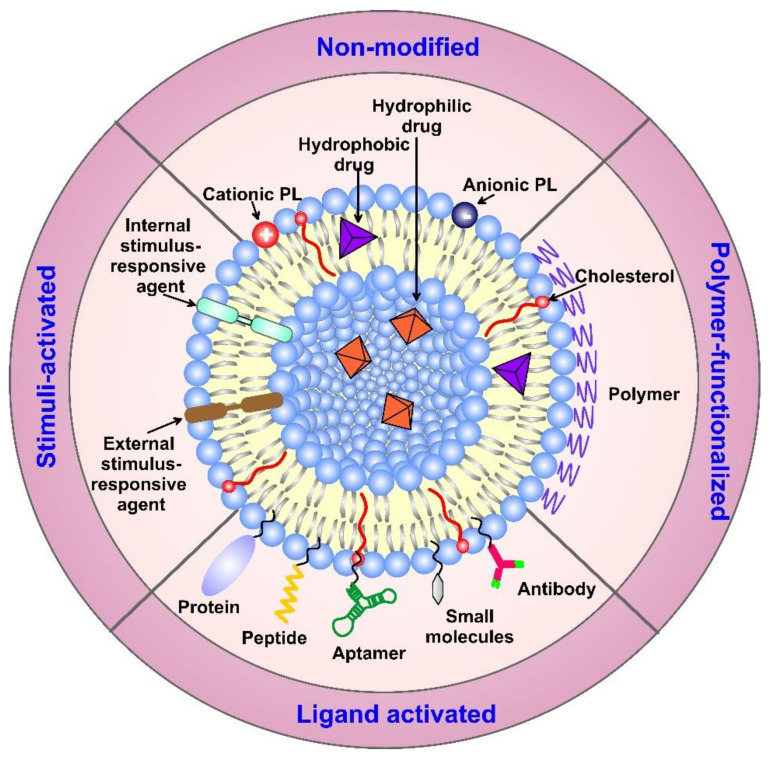
Surface modification strategies of liposomes, together with their classification. The modified carriers can contain active components, such as drugs, small molecules, proteins, and/or targeting moieties, such as antibodies, peptides, aptamers, etc., conjugated on the surface of the vehicles through different linkers, non-covalent or covalent bonds, and electrostatic interactions. Abbreviation: PL—phospholipid.

**Figure 2:**
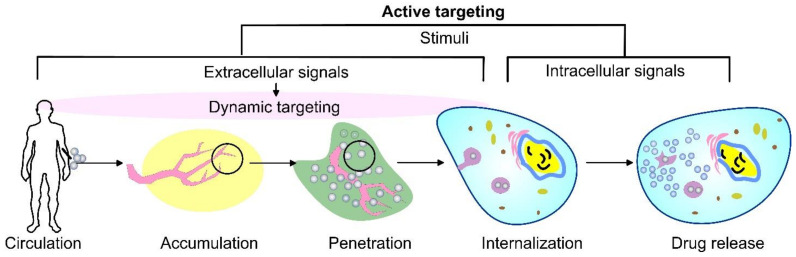
A schematic representation of cancer drug delivery by stimuli-responsive vehicles. Depending on the area of activation, the stimuli can be divided into extracellular signals that focus on dynamic targeting during circulation, accumulation, penetration, and internalization, as well as intracellular signals.

As an alternative, the active targeting of cancer sites uses various ligands to recognize antigens expressed by tumor cells (Figure 1). The targeting of cancer cells may be performed by antibodies or antibody fragments (immune-liposomes), aptamers, charged molecules, proteins, peptides, or other receptor-ligand bindings for site-specific targeting. Relying on the ligands, such as folic acid (FA), CD44 (cell surface glycoprotein), vascular endothelial growth factor (VEGF), integrin, etc., presented by a few types of tumors, the problem of tumor cell heterogeneity in the expression of their surface markers has not been overcome yet. A possible direction for future investigations is the ligand coupling of different internal and external stimuli for more sensitive detection. By attaching various chemical-specific moieties to the liposome’s surface, the system can respond to different physiology-dependent or physical stimuli. Stimuli-responsive liposomes are also considered to be effective in on-demand drug delivery. These “smart” nanocarriers undergo triggered drug release on physiology-dependent (internal) or external (physical such as light, temperature, magnetic field, ultrasound, etc.) stimuli, thus providing better accuracy in the concentration, timing, dosage, and location of release [13]. By modification of the vesicle surface, liposomes can be used both to impart “smart” character and attach ligands for active targeting. As shown in Figure 2, the active targeting of the drug-loaded carrier can respond to both extracellular and intracellular signals. The former dynamically target the liposomes toward the tumor tissue during circulation and trigger their accumulation, penetration, and internalization into the cancer cells [14]. The intercellular signals are responsible for the release of drugs in different cell compartments. Although these smart systems have been extensively explored as pharmacotherapy agents, different adverse effects have limited their clinical applications [15]. Therefore, the selection of proper constituents and activation mechanisms is a crucial factor in engineering the modified drug carriers, since this predetermines their distribution, targetability, and efficiency at specific sites.

Since liposomes are known to be one of the most successful DDS known so far, this review reports on recent developments in responsive drug delivery liposome formulations for cancer therapy by analyzing examples of smart nanovehicle achievements. Although intensively investigated for over 50 years, liposomes are still objects of vigorous research. Unique features, such as design, presence of functional groups, biocompatibility, toxicity, solubility, etc., that make these nanoparticles (NPs) attractive nanomedicine candidates are consistently discussed.

## 2. The State of the Art of Liposomes for Drug Delivery and their Production Methods

### 2.1. Main Characteristics of Liposomes for Drug Delivery

**Figure 3:**
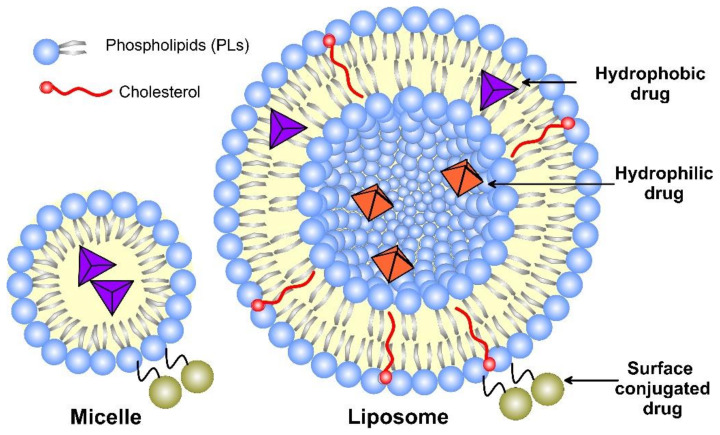
A schematic structure of micelle and unilamellar liposome, together with the possible drug location.

Liposomes, as nanomedicine representatives, have a rapidly evolving design that improves their interactions with cellular targets at the nanoscale. The functional scaffold for the fundamental cell-like kinetic behavior of liposomes that impart surface modification for active and passive targeting is the phospholipid bilayer envelope. Generally, the liposome drug-loaded vesicle consists of a hydrophilic interior part and shell of one or several concentric phospholipid bilayers or lipid monolayer structures, called micelles (Figure 3). Considering the water solubility of the loaded drug, the latter may be bonded to the surface of the vesicle, encapsulated in the aqueous core, or included in the hydrophobic space of the bilayer lipid membrane by Wan der Waals forces [16]. Due to the enhanced lipid–lipid exchange, the dissolution rate and convective flux of the entrapped drug are accelerated. Additionally, by modification of these drug-delivery vesicles, they may be targeted toward specific cells, tissues, or organs [17], while decreasing the systemic side effects.

### 2.2. Factors That Influence the Physicochemical and Drug Delivery Properties of Liposomes

The physicochemical and drug delivery properties of liposomes depend on their composition, surface charge, size, number of lamellae, bilayer fluidity, surface modification for targeting, and production method [11]. These characteristics can be modified and uniquely tailored at a certain stage of the liposome’s production to favor specific biological, chemical, and mechanical properties and drug delivery targeting. The main factors affecting the stability of liposome formulation, their bioavailability, and drug delivery ability are as follows.

#### 2.2.1. Bilayer Composition and Fluidity

There are several types of phospholipids used for the preparation of liposomes, such as natural phospholipids, modified phospholipids from natural sources, semi-synthetic or fully synthetic phospholipids, non-natural head group phospholipids, etc. Natural non-toxic phospholipids, sphingolipids, cholesterol, and hydrophilic polymers are typical constituents of the liposomal bilayer. The membranes of the liposomes used in medicine mostly consist of phosphatidylcholine (PC) and a little amount of phosphatidylethanolamine (PE) present within them both, with neutral charges under physiological pH [18]. Based on the overall charge of the lipid part of the vesicle, the liposomes can be anionic, cationic, or neutral.

The lipid composition and transition temperature (Tc) determine the curvature of liposomes. At Tc, phospholipids shift from gel to a liquid-crystalline phase with greater fluidity. Tc depends on the length of the fatty acid chains and their saturation [19]. Tc decreases with a decrease in chain length and an increase in the double bonds in them. The presence of unsaturated lipids within the liposomes compromises the integrity of the lipid bilayer through lipid transfer to lipoproteins, disintegration, and leakage of the content. Therefore, Tc predetermines the permeability and fluidity of the liposome bilayer. The fluidity of the bilayer is also affected by cholesterol, which increases the fluidity in the core of the bilayer because of its aromatic rings laying parallel to the fatty acid chains, while the viscosity is increased close to the phospholipid headgroups where its hydroxyl group is placed [20]. Cholesterol is used in different liposomal formulations because its presence stabilizes the carriers by protecting them from interactions with different proteins, such as transferrin, albumin, macroglobulin, etc. [21].

#### 2.2.2. Lamellarity and Size

**Figure 4:**
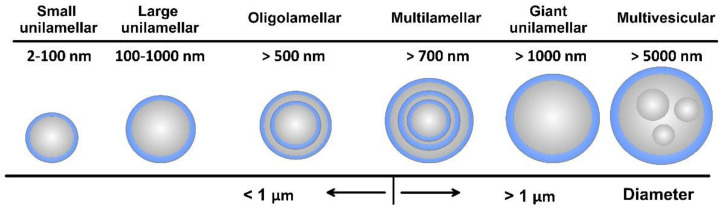
Overall view of liposome types together with their indicative size.

As amphiphilic structures, lipid materials and phospholipids spontaneously disperse in water to form physically stable liposomes, where the constituent lipids are usually not covalently bonded with each other, in contrast to the monomer units that build polymers. When consisting of only one phospholipid bilayer, the liposomes are termed unilamellar, while those containing several bilayers are called oligo- or multilamellar vesicles (Figure 4). Small unilamellar carriers, with sizes less than 100 nm, are usually smaller than multilamellar, but there are also large (>100 nm) and giant (>1000 nm) unilamellar vesicles. The amount of the loaded drug compound and the drug release rate are dependent on the number of phospholipid bilayers. Overall, the liposome size can vary between 20 nm to 2.5 μm, and both the size and number of bilayers determine the amount of the encapsulated drug [22]. As discussed, for injectable clinical applications, the liposome diameters should not exceed 200 nm, so they can be considered submicron or nanostructure carriers.

#### 2.2.3. Surface Charge

The surface charge of liposomes depends on the phospholipid head groups. Negatively charged phospholipids are faster recognized by macrophages than neutral phospholipids that shorten the blood circulation time. Neutral liposomes are stabilized by small negative charges, due to increasing the repulsive electrostatic forces affecting the aggregation-dependent mechanism of phagocytic uptake [11]. On the other hand, cationic liposomes undergo opsonization (interaction with plasma proteins) that triggers phagocytic-mediated clearance by the liver, spleen, and lung. Because the uptake of positively charged liposomes appeared to be higher than that of negatively charged, most of the FDA-approved liposomes are negatively charged [19]. Moreover, cationic liposomes also hinder interactions with tumor cells, and their accumulation in tumor stroma just performs the function of a drug-loaded depot.

#### 2.2.4. Stability and Bioavailability

To predict the bio-behavior of a potential nanocarrier in the body, the protein adsorption (the so-called protein corona) on the liposomal surface should be considered. Since changing the liposomal biological identity, the absorbed proteins determine the organism’s response, including the cell uptake body distribution and clearance [23]. Even liposomes that are synthesized from natural phospholipids are recognized as foreign particles in the body and cleaned by the mononuclear phagocyte system [24]. By choosing either natural or synthetic (phospho)lipids as ingredients, the lifespan, biocompatibility, and biodegradability of a liposome could be changed. 

The first (conventional) generation of liposomes that load drug molecules to their unaltered surface faces (Figure 3) challenges, due to its inherited instability. Their major shortcoming includes the lack of fast and easy preparation routes, rapid decomposition in the organism before achieving the therapeutic effect, low degree of drug-loading capacity, and instability in the bio-environment [25]. The next-generation liposome formulations overcame the tendency to fuse because of high surface tension and escaped unspecific plasma protein adsorption by coating them with polymers, such as PEG (called “stealth liposomes” with a size less than 200 nm) [26] or super-hydrophilic zwitterionic polymers [27]. Hydrophobic long-chain polymers, such as PEG and glycolipids, are known to prevent rapid clearance and increase blood circulation time [28]. Liposome encapsulation reduces drug clearance by the immune and renal systems and increases their availability in the organism [29]. It was found that PEGylated small-sized (100–150 nm diameter) liposomes showed fewer interactions with opsonin [30], thus reducing their consumption by the reticuloendothelial system. The long-circulating “stealth” liposomes are found to target the cancer cells by the EPR mechanism, thus decreasing the drug toxicity in the organism. PEG encapsulation was successfully proven in the FDA-approved nanomedicine Doxil^®^ [29]. Additionally, a variety of affinity ligands, such as peptides and antibodies, can be immobilized to the liposome formulations with PEG linkers for targeting the disease cell [31].

Hybrid liposomes consisting of a solid organic or metal oxide core and lipid shell and characterized by good size, morphology, mechanical stability, and drug-release kinetics have been proposed [32]. The lipid shell reduces drug diffusion, limits water penetration across the interface, and mimics the biological membrane. Recently, for stability enhancement and suitable drug encapsulation, solid lipid NPs, instead of liquid lipids, in the preparation method have been proposed. Such NPs demonstrated biocompatibility, biodegradability, acceptable bioavailability, higher shell life, improved drug targeting release, absorption, and dissolution, as well as easy large-scale production and sterilization [33]. However, the loading capacity of hydrophilic drugs is limited. Higher loading capacity, with a wider range of drugs, was obtained in the solid and liquid phases of lipids with imperfect crystalline structures [34]. When hydrophilic drugs are loaded by a covalent bond to hydrophobic molecules, resulting in the formation of salt, the lipid-drug conjugate can protect sensitive drugs from the acidic stomach conditions, while a polymer (such as PCL and PLGA) –liquid hybrid NP can form a core-shell structure when conjugating with drugs [35]. When delivering lipophilic drugs, the latter is maintained in its solubilized form in the lipids and by using lipid excipients, such as triglycerides, mixed glycerides, polar oils, surfactants, and co-solvents, and various favorable reactions, such as improved bioavailability, antioxidant effect, topical delivery, enhanced drug therapeutic effect, etc., are observed [36]. In developing such lipidic products for poorly aqueous soluble drugs, the issues related to difficulties in the manipulation and weak stability of lipid formulations can be successfully unraveled. To further enhance the therapeutic efficacy, liposomes with stimuli-responsive drug release have been developed. 

### 2.3. Production Routs of Liposomes

**Figure 5:**
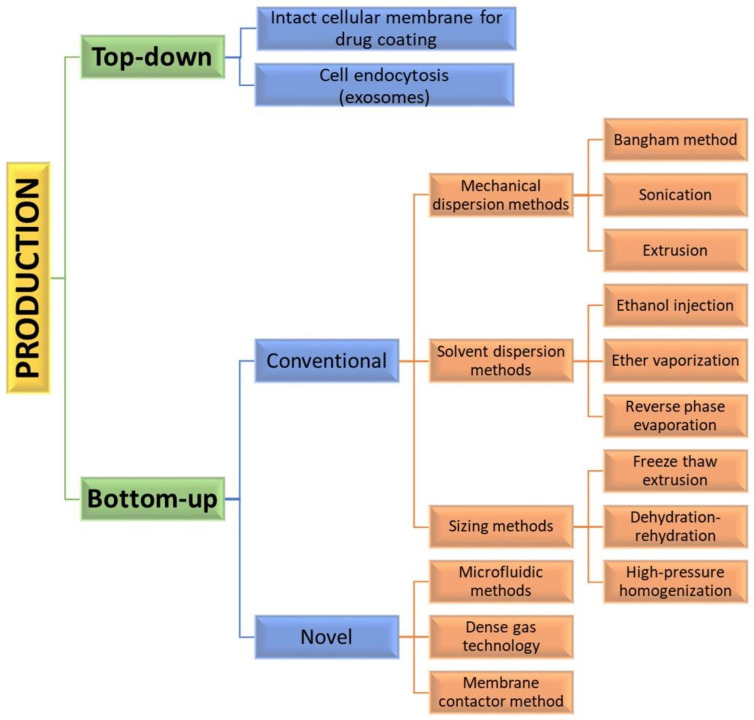
Diagram of various manufacturing engineering approaches and processes used for the synthesis of liposome vesicles.

Assembly methods play an essential role in liposome characteristics, including drug encapsulation efficiency and drug release profiles. Both “bottom-up” and “top-down” engineering approaches have been used to form individual small vesicles (Figure 5). Numerous synthetic liposomes-like DDS with finely tuned physicochemical properties were synthesized through “bottom-up” processes, but it is still hard to achieve complex functionalities. 

Natural cellular membrane-derived vehicles made by “top-down” techniques inherit their natural functionalities, or even enhance them, by using genetic modification. Similar to the mammalian exosomes that are released in the extracellular space, exosome-bound tumor antigens were found to induce a more active antigen-specific antitumor response than the corresponding soluble antigens [37]. When the membrane phospholipids are disturbed, they tend to rearrange into small spherical particles composed either of monolayers (micelles) or bilayers (liposomes). Mesenchymal stem cells (MSC) capable of homing to different cancer cells are often used as a source for producing anticancer-targeting vesicles [38]. However, the formation of liposomes is not a spontaneous process. 

The encapsulation of synthetic nanoparticles with cellular membrane can be achieved by their internalization by cell endocytosis and the subsequent release of the vesicle-enclosed particle (exosome formation) [39] or by collecting intact cellular membranes that are afterward used for coating inert or biodegradable particles. Direct loading of exosomes incubated with a certain compound or simple drug mixing with the exosomes has been also reported [40]. For using exosomal carriers in drug delivery, it may be necessary that the exosomal interface be modified by fusing synthetic liposomes and exosomes. In that way, immunogenicity is decreased, while the colloidal stability and half-life of exosomes in the blood are improved [41]. This separate preparation of particles and top-down approach for preparing cellular membranes offers good flexibility and biological stability [42]. The use of natural membranes saves labor-intensive processes, such as protein identification, purification, and conjunction. However, adverse effects on cargoes and liposomes, such as aggregation, could also be expected. 

The “bottom-up” strategies for liposome preparation can be classified into three main groups: mechanical dispersion methods, solvent dispersion methods, and size-adjusting methods. All of them use the precipitation of dissolved lipids into an aqueous solution and because of changed solubility, liposome formations occur spontaneously. The mechanical methods include the Bangham method, which produces liposome formations via thin lipid films deposited by organic solution on the glass surface by shaking at temperatures higher than Tc [43]. After that, the solvent is removed, and the lipid film is hydrated, while agitating to the lamellas from the surface to form spherical structures with heterogenous micron sizes. Another mechanical approach is sonication under a passive atmosphere with a bath or a probe sonicator to obtain liposome carriers with diameters down to 15–25 nm [44]. By applying dual asymmetric centrifugation, the mechanical turbulence and cavitation produce nanoliposomes with a size of around 60 nm and homogenous size distribution but poor productivity [45]. A third mechanical dispersion method is membrane extrusion, which consists of extrusion above the phase transition temperature through polycarbonate pore-containing membranes, allowing the formation of liposomes with dimensions close to that of the membrane pores’ size. The method is simple and reproducible in downsizing, but sensitive to product losses [46]. 

The solvent dispersion methods include ether vaporization and ethanol injection routes. The former consists of slow ether injection to a mixture of lipids into a warm aqueous solution. As a result of ether removal under vacuum and heat, unilamellar carriers with good size distribution and higher volume trapping activity liposomes are formed. During the ethanol injection, the lipids dissolved in the organic phase are injected into aqueous media, thus forming liposomes. However, since some liposomes are poorly soluble in ethanol, adequate mixing is not achieved. Additionally, the liposome population is heterogeneous, while alcohol removal is difficult. The presence of residual solvents in the bilayer can change the physical and mechanical characteristics of the membrane [47]. However, solvent injection methods are suitable to become continuous production routes. The alternative solvent dispersion method is reverse-phase evaporation, where different phospholipids and cholesterol can be used. The lipids are dissolved in an organic solvent, where inverted micelles are produced and shaped by sonication in a mixture of a buffered aqueous solution. The water-soluble molecules are encapsulated into liposomes, and the slow elimination of the organic solvent converts the micelles from viscous to gel form. The aqueous volume-to-liquid ratio in these formulations is high, making them suitable for entrapping a large percentage of aqueous material. However, the encapsulated compound is in contact with the organic solvent and has brief sonication periods, which makes the process unsuitable for fragile molecules, such as peptides or DNA strains [26].

The sizing methods include freeze-thaw extrusion, which creates large unilamellar vesicles, due to the fusion of small unilamellar liposomes during repeating cycles of freeze-thaw and vortexing the sample. Similarly, during the dehydration–rehydration technique, small unilamellar liposomes in the buffer are mixed with the moiety to be entrapped and then freeze-dried. After rehydrating the vesicles, larger formulations are constructed because the frozen phase becomes more concentrated and flattened. Since heterogeneity of the size is observed, sizing by sequential extrusion at low pressure through polycarbonate membranes or gel-permeation chromatography [48] can be applied. The size reduction follows the mechanism of rupture at the entrance of the membrane pore and rearrangement during the membrane passage. During the high-pressure homogenization technique, the liposome suspension is passed through a narrow gap under high pressure and broken down by the cavitation, turbulence, and shear force of the velocity gradient and, after that, re-arranged into smaller liposomes. By increasing the pressure and process cycles, the polydispersity and particle size decrease, which results in decreased encapsulation efficiency [49]. 

All these production routes are characterized by some disadvantages, such as the need for a large amount of organic solvent, poor drug loading efficacy, low yield, and time-consuming issues [50]. Bulk methods also produce products that are not uniform in size and lamellarity due to poor control over the chemical and mechanical conditions of the process. Additionally, these techniques may not be suitable for processing various biomolecules that can undergo structural changes [50].

By applying precise control of fluids in a constrained volume, novel microfluidic methods offer the ability to remove the organic phase from the final product, a high degree of control over the production route, and reproducibility in the production of monodisperse vesicles [51]. By using microfluidic systems, many factors, such as osmolarity, pH, temperature, vesicle size, salinity, and fluid mechanical forces, can be precisely controlled. Such methods are pulse jetting [52], ice droplet hydration [53], hydrodynamic focusing [54], hydrodynamic pinch-off mechanism [55], solvent extraction-based droplet microfluidics [56], etc. The main disadvantages of microfluidic systems include the use of low quantities of solution, resulting in a low volume of the manufacturing process and rather clumsy methods for establishing and operating. Another new method of liposome formation namely dense gas technology, employs supercritical fluids, such as supercritical carbon dioxide, that are excellent solvents for many lipids; after mixing with the water phase, liposomes with narrow size distribution are synthesized [57]. The next modern production route is the membrane contactor method, in which a lipid phase dissolved in alcohol is pushed through a porous membrane into an aqueous phase flow, where lipid molecules are self-assembled into homogenous-size liposomes [58]. All these modern methods have high scaling-up abilities, allowing for large-scale liposome production, but until now, the disadvantages of these novel techniques were mainly connected with their high capital cost [50], which circumstances can discourage their industrial development. Furthermore, strict control over quality, purity, on-shelf stability, and sterility is required by pharmaceutical regulations, which can represent a limit to efficient technology transfer. 

### 2.4. Drug Encapsulation Techniques

Methods for encapsulating different drug agents within liposomes are either *passive* when the cargo is encapsulated during liposome formation or *active* when the loading follows the formation of empty liposomes. Hydrophobic drugs can be directly combined into liposome formations during carrier formation, and the trapping effectiveness depends on the solubility of the drug in the liposomal membrane and may reach 100% [26]. The passive encapsulation efficacy depends on the aqueous volume enclosed by the vesicle, which is proportional to phospholipid concentration in the dispersion and morphology of the vesicle. 

Water-soluble drugs are usually actively entrapped by employing, for example, the blending of empty liposomes with a concentrated drug solution that distributes equally by diffusion [59]. The method that creates diffusion gradients is called “remote loading”. For increasing the loading effectiveness, pH gradients across the bilayer or ion gradients can be used [60]. Transmembrane proton gradient can be generated by preparing liposomes in low pH buffers or by incorporating ionophores that couple the outward movements of mono or divalent cations with the inward movement of protons, thus acidifying the liposome interior. Another route is the preparation of liposomes in the presence of a weak base, such as ammonium sulfate. The removal of the external ammonium salt generates a pH gradient that helps the drug-loading process. When loading two different drugs in the same liposome system, a combination of passive and active encapsulation could be applied. For example, cytarabine is passively loaded into the liposomes when hydrating the lipid foams and after sizing, and a daunorubicin buffer solution is incubated with the cytarabine-loaded liposomes. Daunorubicin diffuses through the lipid bilayer and is actively accumulated inside the liposome, due to the copper gluconate/triethanolamine-based loading [61].

An efficient strategy for loading drugs within liposomes is covalent linkage. For example, muramyl tripeptide-phosphatidyl ethanolamine (MTP-PE) was linked with a peptide spacer—a formulation that had an improved lipid solubility, rather than muramyl dipeptide itself (a component of the cell wall of Gram-positive bacteria), has been used for loading drugs within liposomes, such as in Mepact. These amphiphilic molecules were able to intercalate into the phospholipid membrane during liposome synthesis, and no free MTP-PE existed [62].

## 3. Approved Liposomes and Drugs for Loading

Liposomes have become the first generation of nano drugs approved for anticancer treatment [63]. Many liposomal formulations have been developed and are now available on the market. The first FDA-approved nanomedicine (Doxil^®^), in 1995, to treat ovarian cancer and AIDS-related Kaposi’s sarcoma was Doxorubicine-loaded PEGylated liposomes, and from then on, liposomes were advocated for the therapeutic and diagnostic needs of various diseases, such as breast cancer, macular degeneration, leukemia, hepatitis, etc. [64]. Non-liposomal doxorubicin hydrochloride drug blocks cell division by interacting with topoisomerase IIα but simultaneously causes cardiotoxicity because cardiac muscle interacts actively with the positively charged Dox [18]. As compared with free Dox, in phase III trials, Doxil^®^ reduced neutropenia (4 vs. 10%), vomiting (19 vs. 31%), alopecia (20 vs. 66%), and cardiotoxicity (3.9 vs. 18.8%) [65]. However, because of their small size and intravasation through the vasculature of diseased and healthy tissues, encapsulated Dox in liposomes showed certain side effects, such as stomatitis, mucositis, and hypersensitive reactions [66,67]. Another approved liposomal anticancer drug DaunoXome^®^ (liposomes loaded with daunorubicin) is applied against AIDS-related Kaposi’s sarcoma [68]. Subsequently, many formulations have become available for cancer treatment, and many clinical trials are still in progress [30]. For example, thermosensitive liposomes are under extensive study, since they prove to be both safe and sensitive to minor changes in temperature. Up until now, ThermoDox^®^, Celsion corporation, is the only thermosensitive formulation in phase III of its clinical trial. In their phase II trials (assessed efficacy and side effects) and phase III clinical trials, lyso-thermosensitive liposomes (ThermoDox^®^, Celsion corporation) are intended for use on hepatocellular carcinoma, breast cancer, and colorectal liver cancer [69]. These long-circulating nanocarriers were clinically combined with radiofrequency ablation to remove the inner tumor part because, at elevated temperature to about 42 °C by radiofrequency ablation, the heat-activated lipid components undergo a gel-to-liquid transition that makes the liposome more permeable for the drug. For that reason, the drug concentration in tumors was found to be 25 times that of the intravenously treated area by Dox [30]. In a phase I trial, to induce a highly localized hyperthermia in liver tumors and trigger the Dox release of ThermoDox^®^, an extracorporeal focused ultrasound enhanced the delivery of systematically circulating liposomal formulations [70].

Other Dox-conjugates targeted towards Her2-antibody overexpressing cells in the presence of trastuzumab showed an increase in drug delivery and advanced the local therapy of metastatic breast cancer during the phase II trial [71]. An interesting liposomal formulation developed by Medigene, Endotag-I, consisting of cationic and neutral lipid formulations of paclitaxel (PTX), interacted with the negatively charged endothelial cells required for cancer angiogenesis [72]. By attacking the dividing endothelial cells, the cationic liposomes were found to inhibit pancreatic cancer development under phase II clinical trials, showing prolonged survival rates when used along with gemcitabine. In phase I trials for the treatment of advanced solid tumors, sphingosomal formulations, composed of cholesterol and sphingomyelin, were found to be good drug-encapsulation platforms with improved duration of exposure and dose intensity [73].

Recently, among various investigated NPs for nanomaterial-based therapeutics, such as self-assembled proteins, viral vectors, polymer NPs, carbon nanotubes, dendrimers, micelles, etc., liposomes, as sterically stabilized formulations, dominate the clinical landscape with FDA-approved products [74]. Some of the liposomal products that are proven to be beneficial in clinical trials and FDA/EMA-approved for various anticancer applications are listed in Table 1, while Table 2 tabulates an overview of the recent patents for targeted liposome formulations.

## 4. Cancer Disease Treatment and Targeted Liposome Therapy

Malignant tumor cells display abnormal morphology, growth (neoplasm), and/or functions [84]. Different approaches, such as surgery, hormone therapy, immunotherapy, chemotherapy, radiotherapy, and applying therapeutic vaccines or stem cell transplants, have been used against cancer. However, the side effects of cytotoxic treatments are various and seriously destroy different healthy living cells, including the blood cells forming bone marrow, digestive tract cells, reproductive system, and hair follicles. Some anticancer drugs affect vital tissues and organs, such as the heart, kidney, liver, bladder, lungs, etc. The barriers to drug penetration in solid tumors include heterogeneous vascular supply and interstitial pressure within cells and tissues. Additionally, the efficiency of the distribution of some drugs may be a compromise of physiological parameters, such as low drug stability in body fluids, binding of proteins with drugs that lead to inactivation, drug uptake by the liver and kidney, and urinary excretion.

To increase tumor exposure and reduce adverse effects, such as alopecia, asthenia, edema, neurotoxicity, etc., caused by active drugs against cancer, intensive research on liposome activity has been conducted. In most cases of liposome exploitation, the drug toxicity decreased to about 50% [26]. Simultaneously, liposomes can overcome drug resistance and exhibit efficient antitumor effects [85]. The efficient and targeted delivery of chemotherapeutic molecules is one of the important keys to successful cancer therapy. The active ligand-directed targeting of liposomes ensures rapid drug accumulation in tumor sites by tumor recognition and restricts the biodistribution of the drug in healthy tissues, thus eliminating the systemic side effects on non-target tissues [86]. An active targeted system can also suppress the defense mechanisms of cancer cells that fight against the drug. After that, the effective release payload inside the targeted cell through the disruption of the liposome carrier can be assisted by the abnormal conditions of the cancer tissue. The liposome nanocarrier can fuse to the cell plasma membrane and release the drug, or the drug can enter the cell through pinocytosis or passive diffusion [87]. Simultaneously, some liposomes may directly interact with the cell membrane and exchange lipids or enter through endocytosis. The endosomes can be disrupted by the liposome carriers and release their cargo to the cytoplasm or undergo maturation with the gradual acidification of the lumen [88]. Therefore, when localized at tumor sites, there are promising alternatives for maximizing the benefit of drug-loaded liposomal use.

The tumor microenvironment contains different factors responsible for tumor cell growth, cancer angiogenesis, and the inhibition of immune response [89]. When compared to normal cells, tumor tissue can have a high temperature (40–42 °C), low pH (6–6.5), high glutathione (reductive) concentration, and overexpression of specific enzymes, such as cathepsin, matrix metalloproteinases, etc. This intrinsic characteristic of tumor microenvironments enables the creation of multi-stimuli responsive liposomes at chronic disease sites for a specific activity. The tumor targeting can focus on overexpressed surface molecules, self-antigens, or specific peptides on cancer cells, including those assisting cell penetration. Although, with narrow limits and igniter-individual variability, such internal stimuli can be used for tissue-targeting liposomes. Simultaneously, utilizing temperature, ultrasound, or pH-sensitive compounds with the liposome formulation is a method to achieve precise drug-release control [90]. Overall, such advantageous liposome characteristics promote an enormous scope for innovations and discoveries in the field of cancer disease. The combined treatment of drug-loaded liposomes with certain local stimulus activations promises to be an effective way of providing potentially increased drug uptake by tumors. This is because single-drug therapy overcomes, with great difficulty, the multi-defense mechanisms of cancer against different external attacks. Such systems for combined treatment enable drug delivery to move beyond biodistribution and pharmacokinetic mechanisms and simultaneously kill tumor cells relying on multiple mechanisms of attack, which will certainly increase the probability of a cancer cure.

## 5. Internal Stimulus-Responsive Liposomes

In today’s generation, advanced drug delivery systems are aimed at targeting the drug in the exact place and observing how effectively the drug is being released therapeutically. However, the ability to control drug distribution and the site of the release from drug delivery systems yet remains to be an unchallenged task; hence, researchers are working on various parameters that are available to manipulate. To achieve a synergistic effect, the chemotherapy can be combined with stimuli from the tumor microenvironment (internal stimuli) to develop a combination cytotoxic therapy. The physiological differences in the biological milieu of tumor sites are used as triggers for payload release. In that way, a self-regulating system that can respond to different biological signals or pathological profiles and modulate its drug release profile can be obtained.

**Figure 6:**
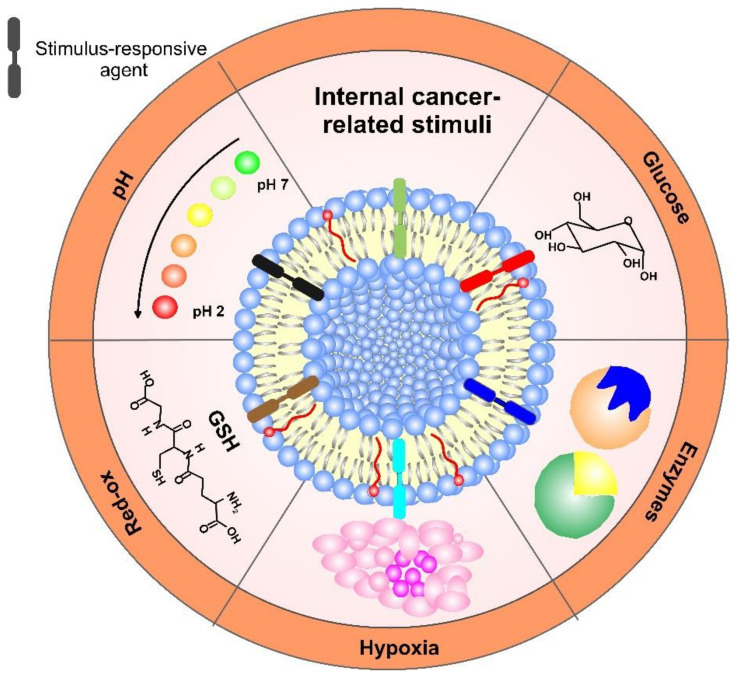
Internal cancer-related stimuli used for the stimuli-triggered release of drug-loaded liposomes.

Different chemical modifications have been introduced in recent years to develop endogenous responsive liposomes for the tumor microenvironment. To accomplish internal stimuli-triggered release or endosomal/lysosomal escape, tailored sensitive molecules that respond to various environmental conditions, such as mild acidic pH (6.5–6.9), the presence of specific enzymes, or hypoxia, have been incorporated in liposomes for drug delivery and, especially, for improved chemotherapy treatment. A schematic overview of the internal cancer-related stimuli used in the construction of liposome formulations is shown in Figure 6. These DDS can expose active stimulus-responsive molecules on cue and release simultaneously or sequentially different drugs, thanks to penetration enhancers. As multifunctional constructions, they can also use multi-release mechanisms, thus providing better efficiency of a given therapy and higher site accumulation of the drug in the organism.

### 5.1. Enzymes-Activated Liposomes

The enzyme-responsive liposomes are promising nanotherapeutics because of their selectivity and specificity. Enzyme-responsive liposomes release their cargo upon contact with the enzyme through several destabilization mechanisms: (a) structural perturbation in the lipid bilayer, (b) removal of a shielding polymer from the surface and increased cellular uptake, (c) cleavage of a lipopeptide or lipopolymer incorporated in the bilayer, and (d) activation of a prodrug in the liposomes [91]. Enzyme-responsive liposomes were developed via a modular approach, exploiting the synthetic lipid switches containing variable enzyme substrates that, when removed, yield the decomposition of a self-immolating linker producing a non-bilayer lipid that perturbs the membrane and triggers the release of contents enabling the targeting of a range of enzymes that are overexpressed in diseased cells for drug delivery applications. The utilized enzymes for enzyme-mediated drug release can be either extracellular or intercellular.

Among the many enzymes used to aid drug delivery to cancer, proteases are promising and widely explored agents, since they are overexpressed in cancer tissue. Substrates of these enzymes can be used as ingredients in liposome vehicles for achieving enzyme-mediated drug release. Such substrates for matrix-metalloproteases 2 (MMP2—a class of extracellular Zn-dependent endopeptidases) cleavage are short peptide linkers between TAT-functionalized drug-loaded liposomes and PEG chains [69]. After cleavage of this linker, the drug-loaded vehicle was exposed to the target site and subjected to TAT-mediated internalization. Zhu et al. synthesized a PEG-lipid conjugate sensitive to extracellular MMP2 with anti-nucleosome monoclonal antibodies for active targeting [92]. The pharmaceutical nanocarrier provided enhanced cellular internalization via cell penetration in a tumor microenvironment. Such nanocarriers were successfully used for siRNA delivery, demonstrating near 70% gene-silencing activity in tumor-bearing mice [93].

Self-assembled different homologous of PEG-phosphoethanolamine (PEG-pp-PE) copolymer also indicated MMP2-sensitive drug delivery to cancer cells. The PEG–peptide–lipid structure, and the balance between hydrophilic and hydrophobic segments of the copolymer were pivotal for the inhibition of P-glycoprotein-mediated drug efflux [94]. Recently, multifunctional vinorelbine plus dioscin liposomes developed with cleavable peptides as a linker for long-chain PEG showed enhanced active targeting and cellular uptake via electrostatic adsorption after being hydrolyzed by MMP2 enzymes. The targeted liposomes had obvious accumulation in tumor sites and magnificent antitumor efficiency [95].

A lysosomal protease termed cathepsin B was also found to be upregulated in different cancer types, including brain, lung, and colon tumors, thus providing an important advantage for target delivery inside the cancer cells. For that reason, PHEG coats, such as PEG covering the liposome, were used to increase the circulation time of DDS within the bloodstream, while, at the same time, PHEG was prone to degradation not only by Cathepsin B, but also by related proteases, such as pronase E and papain [91]. When incorporated with liposomes, PHEG is degraded by the protease and liposomes and aggregated with other liposomes, causing conformational changes and drug release.

Phospholipase A2 (PLA2) is an enzyme that degrades phospholipids at the lipid water interface and is overexpressed in various cancer types, such as breast, lung, prostate, and pancreatic [96]. The catalytic activity of the enzyme is enhanced when phospholipids are organized as liposomes and are dependent on membrane charge and lipid composition. By finely tuning the level of cholesterol in anionic unsaturated liposomes, Østrem et al. were able to adjust the enzyme specificity based on fluidity. They incorporated cholesterol in PLA2-sensitive liposomes. Such incorporation was not previously possible because of the reduced PLA2 activity. These liposomes loaded with oxaliplatin revealed efficient growth inhibition against two different (colon and mammary carcinoma) cell lines, compared to clinically used stealth liposomes. However, after three days, all mice having received the PLA2-sensitive liposomes were euthanized, due to severe systemic toxicity [97].

Despite their specificity, enzyme-responsive liposomes may suffer from hindered control over the initial response time of the nanosystem, low compatibility between the enzyme and the substrate, and extra-tumoral liposomal breakdown. However, modular strategies’ efficacy can be increased and can be tailored to target different enzymes, providing a promising new avenue for advancing liposomal drug delivery [98].

### 5.2. Red-Ox Activated Liposomes

Glutathione (L-γ-glutamyl-L-cysteinylglycine, GSH) is a reducing agent in which thiol groups neutralize ROS accumulation in cancerous tissues. It protects the biological systems by oxidizing itself to glutathione disulfide (GSSG), which is reduced back to GSH by glutathione reductase. The concentration of GSH in cancerous tissue was found to be about 100 times greater than that in healthy tissue and 100–1000 times higher than that in blood [73]. The redox homeostasis in tumors is distorted with increased ROS (10–100 times greater than in normal tissue [99]) because of mitochondrial and antioxidant enzyme disfunction and overexpression of NADPH oxidases. The increased levels of ROS are decisive for the remodeling of the extracellular matrix, increased cellular DNA damage, amplified rate of mutations, and tumor progression [100].

**Figure 7:**
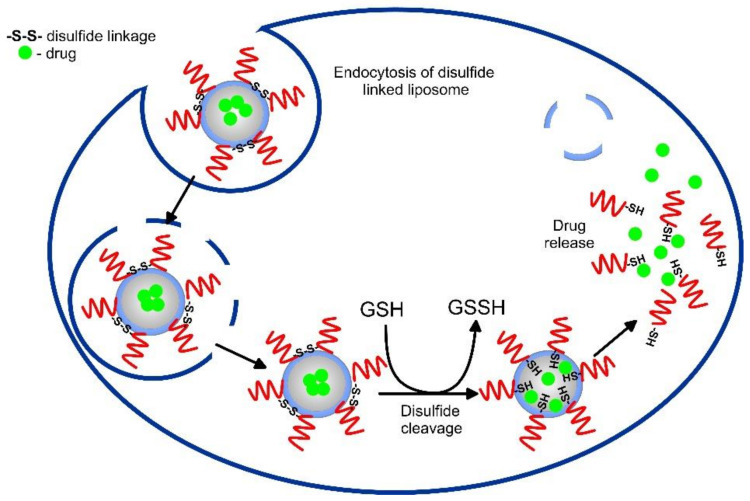
Schematic mechanism of drug release of redox-sensitive liposome that undergoes endocytosis, cleavage of the disulfide linkage at a high level of GSH, decomposition of the bilayer, and subsequent release of the encapsulated drug.

The elevated GSH levels in cancer cells, compared to healthy tissue, present a prominent stimulus for stimulus-dependent drug delivery. Decorated DDS with redox-sensitive bonds or linkers, such as disulfide bonds that are known to cleave by GSH, liposomes can deliver drugs in the intercellular compartments and tumor sites (Figure 7). The disulfide bonds are reduced to thiol groups after endocytosis inside the tumor cells, where a higher level of GSH is observed and the nanostructure dissociates, thus releasing the encapsulating drug [101]. Noyhouzer et al. used ferrocene-modified unilamellar phospholipid liposomes as DDS for controlled payload release by a redox-activated mechanism inside the HeLa cancer cells [102]. Since ferrocene groups on the surface triggered redox reactions, the flow cytometry evaluation of drug release showed 200-times stronger signal for the modified liposomes, indicating higher specificity to the cancer cells. Irinotecan (IR)-encapsulating redox-responsive liposomes, based on disulfide phosphatidylcholine, PEG2000, and cholesterol, and an average size of 125.5 nm, were developed by Wang et al. [103]. Their results indicated the ultra-high loading capacity of the nanocarriers, GSH breakage of the disulfide bonds, and superior pharmacokinetic antitumor efficacy, compared to free IR and conventional IR liposomes.

Recently, Mirhadi and his group used an organoselenium (10,10′-diselenediylbis decanoic acid (DDA)) redox-sensitive compound to enhance the therapeutic performance of Dox-loaded liposomes [104]. The optimum formulations indicated a 30% burst release in the presence of 0.1% hydrogen peroxide at pH 6.5 and efficiently inhibited C26 tumor cells, among other formulations. Coated with ligands, such as hyaluronic acid (HA), to direct the DDS to overexpressed CD44 receptors in A549 tumor cells, an enhanced synergetic antitumor effect was reported [105].

The use of redox stimulants is a sensitive and promising approach for engineering responsive liposomes. Nonetheless, the heterogeneous nature of tumor cells may not be allowed to achieve of specific redox reactions for all cancerous tissues. Additionally, finding tunable redox-active triggers for multidrug delivery at different times and rates presents a challenge for these redox-delivery systems.

### 5.3. pH-Responsive Liposomes

**Figure 8:**
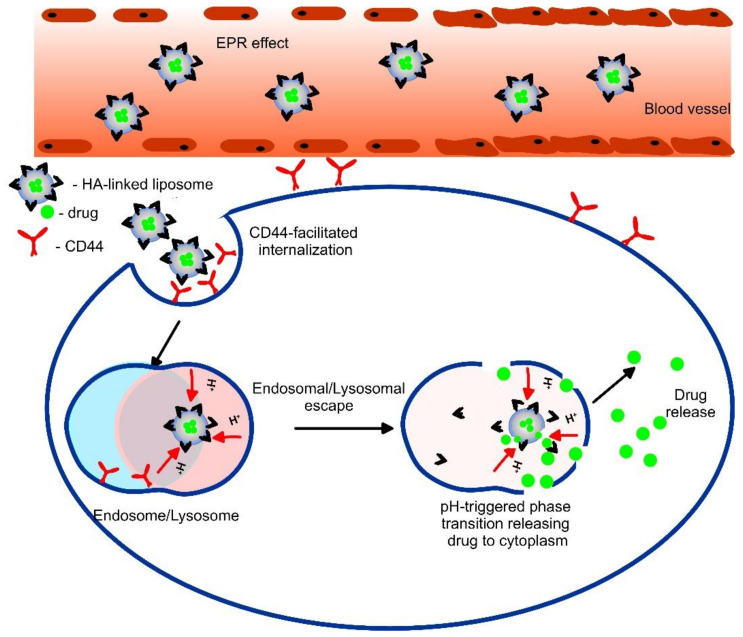
After leaving the blood vessels because of the EPR effect, HA-modified pH-sensitive liposomes undergo CD44-facilitated internalization. At slightly acidic pH in endosome/lysosome, the acid-sensitive moieties undergo protonation, leading to disruption of the liposome and release of a drug inside the tumor microenvironment.

Altered pathological conditions that are observed during tumor progression include substantial pH changes from physiological (pH 7.4). In many tumors, the extracellular pH values are found to be acidic (ranging from 6.5 to 7.2) because of the high glycolysis rate [106]. These low pH values serve as a stimulus that aids the site-specific drug release of liposomes via binding and reactivity inside the tumor interstitium, thus ensuring high cell-kill selectivity. pH-sensitive vehicles usually contain surface-located polymer with acid-sensitive bonds that undergo dissociation in response to pH changes, thus releasing the therapeutics to the cancer tissue and reducing toxicity to healthy living cells. Some polymers could destabilize the phospholipid bilayer, while others can cause fusion of the liposome with endosome/lysosome membrane [107] because of the pH gradient (due to acidification of endosome on fusion with lysosomes) existing within the organelles at the intracellular level (Figure 8). Usually, the structure of the pH-sensitive liposomes includes a phosphatidylamine derivative, pNIPAM (poly(N-isopropyl acrylamide))-based co-polymers, or a weekly acidic amphiphile, such as cholesteryl hemisuccinate (CHEMS), in which the negatively charged group destabilize in the acidic environment triggering fusion with the cell or endosomal membrane and payload release [108]. A formulation with a better pH response, compared to the liposomes of a phosphatidylamine derivative, was found to be lipid diolein with CHEMS containing egg PC and Tween-80 [109].

Fan et al. compared the pH-sensitive response of liposomes conjugates with CHEMS, oleic acid, linoleic acid, and the fundamental lipids cholesterol and phosphatidylethanolamine and found that, since CHEMS has a cholesterol-like structure, it stabilized the phospholipid layer in neutral conditions and had a better pH response in acidic conditions because of its special steroidal rigid structure [110]. However, the acidic environment in perivascular regions can be far away from the bloodstream or the variation in pH can be small, so both can lead to a lack of response from pH-sensitive liposomes [111]. Additionally, since the pH range of the tumors was small (about 1 pH unit), the engineered liposomes were usually found to be incapable of efficient pH response [112].

One useful technique applied to increase the effectiveness of pH-sensitive liposomes was the incorporation of different site-specific ligands. If decorated with targeted ligands, such as peptides or antibodies, the pH-responsive carriers can bind to their ligand cells, resulting in DDS internalization (Figure 8). These ligands, such as hyaluronic acid (HA), folate, transferrin, TAT, RGD, etc., recognize and bind to a specific receptor overexpressed on the target cells, thus triggering endocytosis and endosomal localization. For instance, pH-sensitive (H7K(R2)2)-peptide-modified coumarin-6 liposomes encapsulating Dox were tested both in vitro and in vivo against rat glioma (C6) cells, human glioblastoma (U87-MG) cells, and orthotopic tumor-bearing nude mice, respectively. The formulation showed faster Dox release in pH 5.5–6.5 than in pH 7.4, good tumor-controlling capacity, over 80% efficiency in drug release at a pH of 6.5, and in vivo antitumor anti-angiogenic activity [113]. IgG class antibodies were also extensively studied in pH-sensitive immuno-liposomes [114]. Hyaluronic acid-modified, pH-sensitive liposomes with targeted properties for cells expressing CD44 were developed by Miyazaki et al. [115]. By comparing the pH response of both 2-carboxycyclohexane-1-carboxylated (CH-ex) and 3-methylglutarylated (MGlu) units introduced to hyaluronic acid (HA), it was demonstrated that CH-ex-HA-modified liposomes derived their Dox content into CD44-expressing cells more efficiently than unmodified, only HA-modified, or MGlu-HA-modified liposomes.

Another strategy was focused on increasing the stability and cellular uptake efficiency of pH-sensitive liposomes by surface modification with novel materials, such as maleimide [108], to escape the total degradation after endocytosis when delivered to the lysosomes in cancer cells. Similarly, using polydopamine-coated, pH-sensitive liposomes improved performance, compared to free drugs at pH 6.8–7, which was demonstrated for the modified carriers with 5-fluorouracil (5-FU) cargo. [116].

It follows that pH-responsive liposomes can enhance cancer treatment by precise control over drug delivery to the desired sites. Additionally, for the treatment of cancer with such liposomes, the requirements for drug doses will be lower; therefore, the side effects will decrease which holds great promise.

### 5.4. Hypoxia Activated Liposomes

Because of the rapid tumor growth, there are some tumor regions where the oxygen concentration is significantly lower than that in healthy cells. In these altered environments, cancer cells are found to change their metabolism, accumulating reducing agents, such as NADP and NADPH, alkaline phosphatase, cytochrome P450 reductase, azoreductase, etc. Consequently, the changed redox potential in oxygen-deprived cells can be used as a stimulus for the construction of smart responsive DDS. With lower nutrition and oxygen supply, cancer cells in hypoxic zones divide slower, which makes them more resilient to chemotherapies and radiation. Consequently, many attempts have recently been made to develop nanotherapeutics that combat hypoxia. For example, a well-known hypoxia-responsive electron acceptor, hydrophobic nitroimidazole, was used in liposomes to convert into hydrophilic 2-aminoimidazole under hypoxia conditions and to deliver loaded Dox to the tumor microenvironment [117]. Nitroimidazole incorporates in the phospholipid bilayer of the liposomes, and its hydrophilic derivates facilitate the disassembly of the DDS for triggering drug release [118]. The positive effect of the use of hypoxia-activated liposomes was confirmed by the Dox-loaded hypoxia-sensitive liposomes containing nitroimidazole, which showed better in vivo antitumor efficacy in a cell-derived xenograft model than the free-Dox or Dox-conjugated liposomes evidenced by smaller tumor volume, prolonged survival time, and body weight gain [118]. However, some hypoxia-activated liposomes showed restricted extravasation in deep tumor interiors because of the low penetration in the hypoxic sub-volumes of solid tumors. Other issues for their clinical use are drug packaging and discharge capacity.

Various hypoxia-responsive pro-drugs have also been developed for hypoxic tumor treatment that, unfortunately, is confronted by issues such as rapid clearance and poor selectivity [119]. Shah et al. designed microfluidics-formulated sphingomyelin-cholesterol liposomes with a size of 95 nm, conjugated with vinblastine-N-Oxide that converts to parent vinblastine under an oxygen gradient. In pancreatic cancer cell lines, both liposomes and vinblastine-N-Oxide were selectively activated by low oxygen levels, but liposomes exhibited higher cell inhibition in organoids displaying hypoxia markers than prodrug-treated cell lines [120]. Testing similar formulation against ES2 ovarian cancer in normal and hypoxic conditions, Shah et al. concluded that, under low oxygen conditions, the IC50 value decreased 9.2 folds, as opposed to the prodrug-loaded liposomes under normoxic conditions, confirming hypoxia activation [121]. Utilizing liposomal nanocarriers to co-deliver both hypoxia-activated prodrugs and enzymes, Zhang et al. [122] discovered that stealth liposomes can act as glucose and oxygen elimination agents by conversion of oxygen and glucose into gluconic acid and H_2_O_2_. These glucose oxidase conjugated liposomes loaded with a hypoxia-activated pro-drug, banoxantrone dihydrochloride (AQ4N), were demonstrated to synergistically inhibit tumor growth in the mouse model of the 4T1 tumor. So far, many studies demonstrate excellent advancements in the delivery of chemotherapeutic drugs by hypoxia-sensitive liposomal formulations. However, some disadvantages, such as low penetration of drug in the tumor, local increase in oxygen level, etc., still have to be overcome.

### 5.5. Glucose-Responsive Liposomes

One of the characteristics of cancer cell development is the increased dependence on glucose uptake that fuels aerobic glycolysis for the enhancement of nutrient signaling and generation of new biomass [123]. The glucose uptake is a result of microvascular density and vascular permeability that is not limited to malignancy and is also seen in some benign tumors.

The applied glucose-responsive materials are glucose oxidase, phenylboronic acid, and different glucose-binding molecules, such as lectins or glucose transporters [124]. Phenylboronic acid (PBA) is among the investigated functional glucose-sensing moieties with good stability and long-term storability, as opposed to protein-based systems [125], without triggering an immune response. With molecules bearing OH groups PBA can form reversible covalent complexes. With an increase in glucose level, covalent PBA-glucose complexation is developed. As a result, a volume phase change transition of the hydrogel matrix occurs, which causes the disassembly of the drug or swelling of the vehicle to different extents and substantial drug release, according to the glucose concentration [126].

### 5.6. Other Physiological Biomolecules Used for Liposomal Activation

The promising strategy for anticancer drug delivery is based on the difference in intracellular and extracellular concentrations of adenosine-5′-triphosphate (ATP). This difference is observed for almost all cell types, but the tumor microenvironment in many cases (such as murine lymphoma and mouse leukemia [127]) has a higher ATP concentration that makes ATP-responsive drug delivery system possible to utilize as tumor-specific, especially by combining it with some other responsiveness. Among the different approaches, ATP aptamers (with a strong affinity to ATP) are the most popular because of their simple modification, relatively short sequences (about 30 bases), and specific response. The ATP aptamer (usually single-strand oligonucleotides with high binding affinity against ATP) can bind to the nucleotides forming the DNA duplex via complementary pairing. This DNA duplex can be incorporated into liposomes, together with a certain chemotherapeutic. For example, the fusogenic DOPE liposome encapsulating the ATP-responsive DNA scaffold with Dox could release the chemotherapeutic through a conformational change from the duplex to aptamer/ATP complex in the presence of ATP. Additionally, the liposome shell was protamine peptide-modified for acidic-triggered fusogenic potential with endo-/lysosomes or ATP-loaded liposomes. The study in vitro and in vivo (MCF-7 cancer xenograft nude mice) demonstrated that extrinsic liposomal ATP promoted drug release from the fusogenic liposomes in the acidic intracellular compartments, due to pH-sensitive membrane fusion, and showed subsequent anticancer efficacy [128].

Table 3 summarizes some recent studies focusing on internal stimuli-activated liposomal formulations.

## 6. External Stimulus-Responsive Liposomes

Spatial targeting of drug release using molecular and environmental signatures is, unfortunately, heterogeneously expressed within target sites, which makes the internal stimuli targeting suffer from poor specificity. For example, the variation of pH range in different tumors or normal cells, expression of stimulus substances, etc., are uncertain. Therefore, the accurate control of drug release in a complex physiological and pathological environment with a physiological (endogenous) trigger at the exact moment remains a challenge.

**Figure 9:**
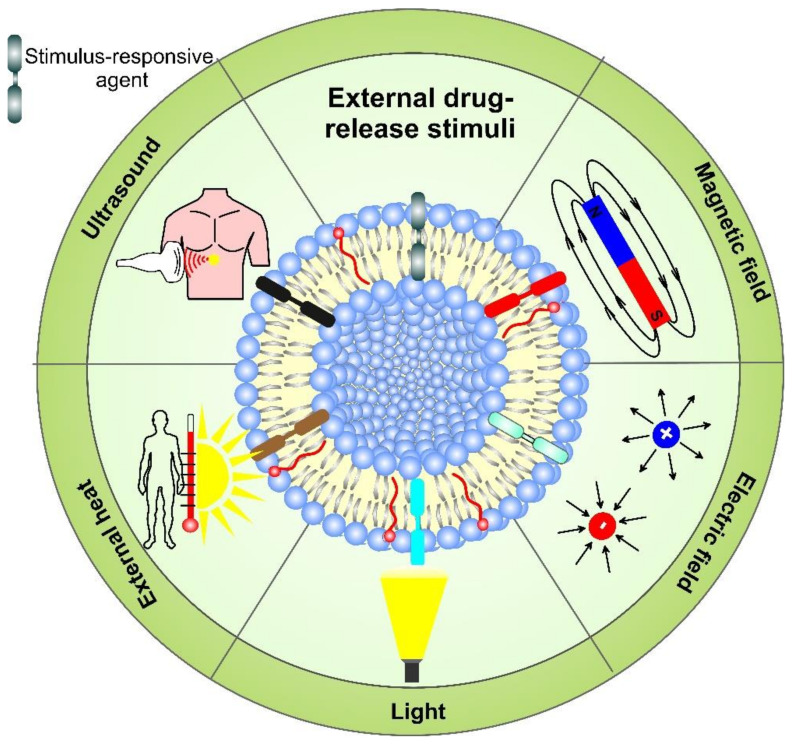
A scheme summarizing different external stimuli for delivery of therapeutics into the cancer cells.

Externally regulated drug delivery liposome systems are indifferent to the tumor microenvironment and the site of action. They can precisely control the drug release profile, depending on the duration or strength of the external stimuli, such as temperature, ultrasound, light, etc. Nanoparticles made of such smart responsive materials offer the unique possibility of designing multifunctional liposome drug delivery systems. A schematic representation displaying the variety of exogenous stimuli used for the therapeutic release application of drug-loaded liposomes is outlined in Figure 9. The dissociation of the loaded carriers can be achieved as a result of light (UV, NIR, far infrared, etc.) irradiation, wave radiation (micro-waves, radio-waves), sound waves (ultrasound), electric or magnetic fields, and temperature changes. The external stimuli are applied to the site of interest to trigger enhanced release from the DDS by destabilization of the liposome structure (e.g., light, temperature, electric field), while the nanovehicles pass the targeted location. The stimulus can facilitate the accumulation of DDS in the target regions by applying an outer force, such as a magnetic field. The multifunctional liposome systems can undergo irreversible or reversible activation/deactivation (e.g., by light) when the triggering exogenous stimulus affects them. In the case of reversible activation, when the stimulus is applied, a certain dose of a drug can be released on demand. However, for metastatic tumors with uncertain locations of the lesions, the application of external stimuli-responsive liposomes can be impractical [90].

### 6.1. Ultrasound-Responsive Liposomes

The ultrasound method is used in many medical applications, such as imaging, tumor and fibroid ablation, dentistry, kidney stone disruption, etc., and this method is considered to be the main method in downsizing the micro range vesicles into a nano size. The two different types of frequencies, low intensity and high intensity, are widely used in the medical field. The low-intensity ultrasound is used in a study for things such as imaging and blood flow studies, whereas high flow intensity is aimed at studying some higher organs, such as kidney stone shattering, tumor/fibroid ablation, etc. Concerning drug vehicles, studies on liposome rupturing, drug release efficiency, and chemical properties of encapsulated drugs revealed that low-frequency ultrasound showed more efficient results [139].

**Figure 10:**
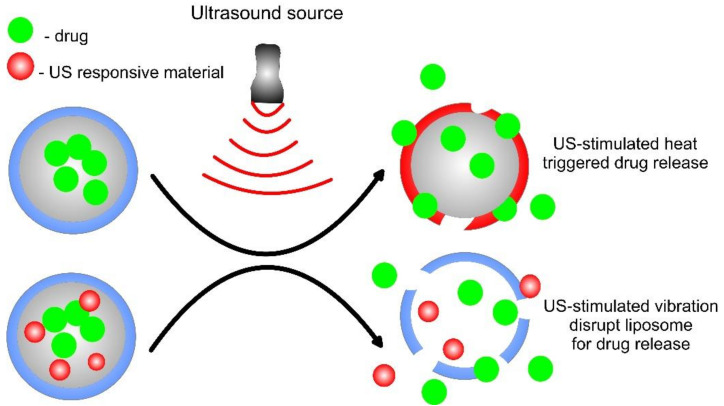
Ultrasound (US) triggered mechanisms of drug releases from liposomes.

Ultrasound waves are characterized by enhanced safety, intrinsic tissue penetration, and spatiotemporal control [111]. These ultrasonic waves/ultrasounds are used to induce either thermal or mechanical effects. The role of ultrasound in the drug delivery of liposomes is to rupture the phospholipid structure by acoustic cavitation (collapse of microbubbles because of oscillating pressure field in the liquid), sonoporation (acoustic cavitation increasing the permeability of cell membrane), acoustic streaming, and hyperthermia. The thermal effects can change vascular permeability, thus enhancing the uptake of liposomes. Ultrasound is widely used in various diagnostic, as well as therapeutic, applications and can penetrate deep into the cells, and nearly the majority of the drug delivery systems work majorly on gas-containing vesicles, such as microbubbles or co-encapsulated within the microbubble. Microbubbles, originally designed as a contrast agent for ultrasound imaging, are the most efficient and well-known ultrasound delivery materials, and they show a lot of advantages, in terms of site-specific delivery and have majorly focused on the release control of drugs/genes. The major molecules used in ultrasound applications generally consist of a poorly water-soluble gas shell of surfactants and lipids, polymers, or proteins. These microbubbles can be prepared using single components or a combination of various molecules, such as lipids, proteins, polymers, or sugars. The mechanisms of ultrasound-triggered drug release are shown in Figure 10. When the microbubble is made of these components, there comes a factor of stability and safety, where stability is defined as the ability of its shell to inhibit the dissolution of the drug into the targeted cells [140]. These microbubbles have a unique property that can dissolve very quickly, and they are made up of various materials, such as perflouropropane or perfluorobutane [141]. With microbubble therapy coming into existence, several researchers have developed a doxorubicin drug-encapsulated AG73 peptide by modifying the liposomes (Ag-73-Dox) that target cancer and endothelial cells, and these help in improving the anti-tumor efficacy by reducing the side effects [142]. However, microbubbles themselves have provided limited drug-loading capacity, of which, the shortcoming can be eliminated by incorporating liposomes encapsulated drugs in which liposomes are conjugated externally to the microbubbles. In that way, a microbubble can carry about 1600 liposomes [143].

Recently, for better results, researchers have focused on designing liposomes containing sono-sensitive materials. For example, porphyrin–phospholipids liposomes were found to be promising as Dox-loaded carriers for sono-dynamic therapy with low-intensity focused ultrasound to release the drug [144]. Hematoporphyrin monomethyl ether is a hydrophobic sono- and photosensitizer, incorporated in the lipid bilayer of liposomes encapsulating vincristine bitartrate, that was found to show excellent antitumor efficiency in vitro and in vivo because of site-specific and time-controlled drug-release [145]. Moreover, constructs of hollow gold nanoshells attached to the surface of robust liposomes and sensitive to both laser and low-intensity ultrasonic stimulation were capable of releasing a small amount of drug on demand in a circulating environment [146]. Vesicles containing bile salts were also found to be sensitive to ultrasound stimuli. Mujoo et al. used bile salts, such as glycocholate, cholate, taurocholate, chenodeoxycholate, and ursodeoxycholate, in the phospholipid bilayers to make the liposomes’ response more sensitive to low-frequency ultrasound. Liposomes containing only DOPE (dioleoylphosphatidylethanolamine) showed high sensitivity to higher-frequency ultrasound than those containing DOPE and taurocholate [147]. Novel ultrasound-responsive liposomes for improved efficacy in rat cancer treatment and reduced side effects were produced by Xin et al. They synthesized mitoxantrone-loaded PLGA NPs and loaded them into liposomes that showed good stability and higher bioavailability than traditional liposomes. Additionally, higher drug release (about 90%) was observed after ultrasound stimulation, compared to non-stimulated liposomes (~50%), due to a change in the fluidity of phospholipids. PLGA NPs vibrated under mild ultrasound stimulation and disrupted the lipid membrane triggering the drug release. A higher elimination ratio after stimulation by the US was also detected [148]. The major advantages of ultrasound-triggered drug delivery are its non-invasive nature, lack of ionization radiation, high penetration depth, and easy exposure. However, some detrimental effects, such as unwanted cell death, irreversible pore formation in the cell membranes, or drug degradation, can be observed.

### 6.2. Light-Responsive Liposomes

**Figure 11:**
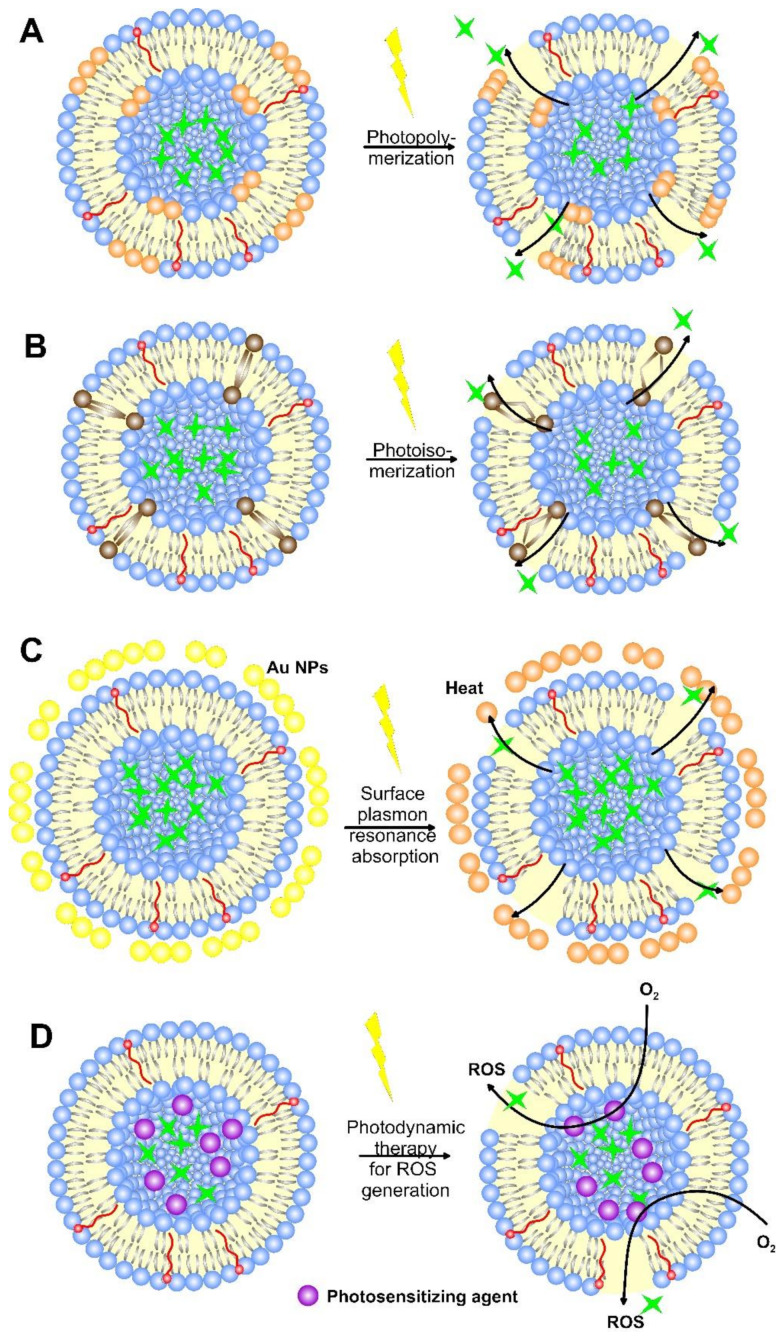
Schematic representation of different mechanisms of drug release by light-responsive liposomes: (**A**) photopolymerization; (**B**) photoisomerization; (**C**) surface plasmon resonance absorption; (**D**) photodynamic therapy for generation of reactive oxygen species (ROS).

Nanoparticle-based radiation therapies were in practice for several decades, which are categorized as photosensitizers (photo-reactive drugs) and the development of photo-triggerable NPs (mostly light-sensitive liposomes) to achieve light-assisted drug delivery as required. Among the external stimuli, light is considered an effective external stimulus, due to its good spatiotemporal control and easy application [149]. Light is considered to be an attractive method for the release of the drug. Various parameters, such as beam diameter, wavelength, duration of light exposure, the intensity of light measured, etc., are considered in controlling the penetration of light inside the human body. There are various examples of light-triggered liposomes that deliver therapeutics at the targeted site in response to different wavelengths of light like ultraviolet (UV), visible, and near IR (NIR). The light-triggered drug release is caused by reversible or irreversible structural modifications of the photosensitized drug carriers, such as phase transition or disruption of DDS, leading to enhanced drug release. A schematic view of the principles of the light-induced liposome drug release mechanism is shown in Figure 11.

The UV and visible light can be used to trigger only superficial tissues (skin, lung, bladder, brain, esophagus), as wavelengths shorter than 700 nm cannot reach the tissues deeper than 1 cm because of light scattering and adsorption by body chromophores, such as lipids, hemoglobin, oxy-hemoglobin, water, etc. [150]. Most of the light-responsive systems respond to UV light that is harmful to cells and has poor tissue penetration, which makes them difficult to bring into practice [151]. Although light irradiation is typically restricted to superficial tissue, nowadays laparoscopy is used for reaching deeper-located tissues. In contrast, NIR-responsive DDS are safer for cells causing less photodamage and displaying good tissue penetration (more than 10 cm) because of limited attenuation within the wavelength of 650–1000 nm [152]. However, the lower energy of NIR may not always induce the desired drug-release response from liposomes.

Many mechanisms have been developed for triggering light-induced drug release by employing light-responsive substances called chromophores (moieties capturing light energy). These mechanisms include photo-crosslinking, photochemical activation, photo-thermal release, and photoinduced cleavage of chemical bonds of specific light-sensitive molecules. Other light-responsive mechanisms include up-converting nanoparticles and two-photon conversion, where a molecule is excited to a higher energy state with two photons with different or equal frequencies that are simultaneously absorbed [153].

#### 6.2.1. Photo-Crosslinking and Photo-Isomerization

The mechanism of photo-irradiation can act as a viable strategy to control the release of drugs externally. Various photo-induced methods, such as isomerization and photo-crosslinking, have been applied to release them from the liposomes. The photo-crosslinking polymerization causes the occurrence of short- or long-lived pores in the phospholipid membranes by polymerizing double bonds in the hydrophobic area of the liposome bilayer (Figure 11A), leading to its local shrinkage and subsequent drug release [154]. Frequently used photo-sensitive groups are o-nitrobenzyl, phthalocyanine, coumarin (which undergoes cross-linking) and spiropyran, spirooxazine, azobenzene, stilbene, and fulgide (which undergo photo-isomerization) [155]. Under UV light, they cause the release of water-soluble molecules/drugs from the liposomes.

Under the irradiation of λ > 310 nm coumarin photo-dimerizes through the formation of cyclobutane bridges, while dimers are reversibly cleaved to monomers by irradiation of λ < 254 nm, which means the process of photo-dimerization and photocleavage takes place reversibly, thus resulting in a photo-promoted drug release [156]. Machácek et al. constructed PC-cholesterol-containing liposomes imparting NIR light-triggered release of Dox, whose rate of release could be altered by varying the amount of photosensitizer (cationic amphiphilic phthalocyanine) to the liposomes, while in the absence of NIR, stable cargo release was maintained [157]. Recently, Meerovich et al. developed a binary drug delivery system consisting of charged (phosphatidyl-I-serine) liposomes and an oppositely charged arginine-rich peptide-photosensitizer conjugate loaded with Dox. The illumination of the binary system containing a phthalocyanine derivate with adsorption of about 685 nm destabilized the liposomal membrane and increased the payload release, which significantly increased the cytotoxicity in a melanoma cell line (B16-F10), compared to a system without illumination [158].

Light-responsive chromophores for photo-isomerization contain a double bond that can undergo *trans*-to-*cis* isomerization in their structure (Figure 11B). The mechanism involves switching from a more stable non-polar and more tightly packed *trans*-isomer to a more polar *cis* isomer upon excitation with UV, visible, or NIR irradiation leading to the formation of a more permeable bilayer and drug release [159]. The conversion is reversible and relaxation back to the *trans*-isomer can be triggered by thermal relaxation or longer wavelength irradiation [160]. Azobenzene is a photoswitchable chemical that undergoes reversible isomerization from *trans* to *cis* at wavelengths between 320–350 nm and reverts to *trans*-form when exposed to visible light (400–450 nm) or heat [161]. Both the steric effect of isomerization and increased polarity of the *cis* form reversibly destabilize the lipid bilayer. It was found that liposomes with up to 25 mol% cholesterol in the membrane release their cargo in response to visible light (470 nm region), in contrast to liposomes lacking steroids [162]. For that reason, Liu and co-authors [163] prepared cholesterol-containing unilamellar liposomes loaded with calcein. They irradiated them every 4 h with visible or UV light the formulations at 37 °C and found that the release rate of calcein increased greatly by UV light, while the visible light completely stopped the drug release. Therefore, the photoisomerization did not influence the liposome integrity and allowed pulsatile drug delivery. Such stimuli-sensitive drug delivery systems can be considered truly “intelligent”.

#### 6.2.2. Photochemical Activation (Amphiphilic Transition)

The mechanisms of disturbance of liposome bilayers and the consequent drug release during photochemical activation include photosensitization-induced oxidation by photo-acid generators, electromagnetic activation, photo-deprotection of fusogenic lipids, etc. For example, plasmenylcholine undergoes phase change under 630–820 nm light irradiation, and when inserted into liposome formulation, this phase cleaved the constitutive lipids to single chain surfactants. When combined with a suitable sensitizer forming singlet oxygen, such as bacteriochlorophyll a, the calcein release from egg lecithin liposomes reached 100% for 20 min, and this drug release was two orders of magnitude faster than that of control liposomes without the sensitizer [164].

In a photochemical activation, using electromagnetic radiation, the activation energy accelerated the reaction; thus, the atoms or molecules absorbing radiation attained a higher energy level and were activated [165]. The technology of photochemical internalization for cytosolic release of lysosome and endosome entrapped drugs can activate lysosomal sequestered sunitinib (antiangiogenic tyrosine kinase inhibitor). The sunitinib, together with the photosensitizer disulphonated tetraphenyl chlorin (TPCS_2a_), accumulated into the membrane lyso/endosomal compartments. The sunitinib-photochemical internalization was evaluated in the human HT-29 xenografts and mouse CT26.WT colon cancer cell lines. In the HT-29 xenografts, only a minor effect on tumor growth delay was observed [166].

In upconverting DDS, the light-sensitive materials convert NIR light to UV light, which is adsorbed by UV-sensitive material that disrupts the liposome. For example, a Dox-loaded liposome coated with amphiphilic co-polymer containing UV-sensitive hydrophobic layer (4,5-dimethoxy 2-nitrobenzyl methacrylate) and outer hydrophilic (poly(methoxy polyethylene glycol monomethacrylate) has been produced. When the micelles were stimulated with NIR light, the latter converted to UV light that was adsorbed by the copolymer, causing hydrophobic-hydrophilic imbalance, disruption of the structure, and Dox release [167].

#### 6.2.3. Surface Plasmon Resonance Absorption (Photo-Thermal Activation)

Different materials, such as Au NPs, MONPs, carbon nanomaterials, melanin, polyaniline, etc., were reported to be able to convert NIR light into heat [168]. Upon exposure to NIR light, the release is accompanied by the photothermal effect that synergies drug action via a photosensitive agent that disintegrates the nanocarrier [153]. For example, when metallic particles, such as gold, are exposed to light, free electrons oscillate in response to an oscillating electromagnetic field of the incident light. The oscillation on the metal surface causes a slight separation of net charges, and a dipole is induced in the direction of the electric field of light. At a specific frequency/wavelength, termed surface plasmon resonance, the amplitude of oscillation reaches a maximum that correlates with a high absorbance of the incident light [161]. The light is scattered when the electron oscillation emits electrons in form of scattered light at the same or shifted frequency of the incident light. For smaller (20 nm) NPs, the extinction was due to absorption, while in 40–80 nm NPs, the scattering contribution increased [161]. Since drug delivery and cell destruction require photothermal effects in which the NPs should adsorb light, smaller NPs are more desirable. The lattice of NPs exchanges the heat with the surrounding very fast (~100 ps) and localized heating imposing thermal and mechanical stresses was observed (Figure 11C). Recently, gold NP-coated liposome loaded with Dox (GCL/Dox) was prepared using thiol-group-spiked liposomes containing glutathione-conjugated gold NP. Over 80% of the encapsulated payload was released from GCL/Dox within 1 h under NIR (660 nm) irradiation. Compared to monotherapy (photothermal effect alone or free-Dox treatment) on the A549 cells, GCL/Dox exhibited a synergetic effect [169]. Similarly, Lui et al. developed thiol-group-spiked liposomes by using GSH containing a poorly water-soluble drug, forming gold nanoshells and NaBH4 as a reducing agent. Excited under NIR light, the nanocarriers promoted cell uptake, compared to those without irradiation, and highly efficient antitumor effects on tumor-bearing mice with an inhibition rate of 83.02% [170]. Although these systems have demonstrated photocontrol by NIR, the process was found to be less efficient than direct excitation on UV light [171]. As a consequence, extended irradiation with high energy density pulse lasers that focus on a small area is required to trigger the disassembly of the photoresponsive carriers, resulting in harmful heating and cell death of healthy tissues. Additionally, issues associated with metal-containing particles such as lower biocompatibility and unintentional particle accumulation are still present. Therefore, photochromic materials that can be controlled with low-intensity one-photon absorption or NIR or visible light are more desired.

#### 6.2.4. Photodynamic Therapy

Photodynamic therapy (PDT) is a treatment based on using light to activate the photosensitizing agents (Figure 11D) that generate radical oxygen species (ROS) or singlet oxygen (^1^O_2_). Those species are highly reactive and can induce damage to both liposomes and tumor cells. Chemotherapy and photodynamic therapy can be combined by embedding both chemotherapeutics and photosensitizing agents to endow liposomes with the function of photo-triggered release. For example, Maier et al. investigated the phototoxicity and cytotoxic mechanisms of 5,10,15,20-tetrakis(meta-hydroxyphenyl) chlorine (mTHPC, Foscan) and liposomal mTHPC formulation against the human and mice-derived osteosarcoma cell lines in vitro. They concluded that the uptake of both mTHPC formulations was higher in tumors than in healthy tissue, while PDT caused significant growth inhibition in both models [172]. Hinger et al. encapsulated mTHPC into lipidots (nanoemulsion) and concluded that, concerning tumor destruction, Foslip was superior to lipidots and Foscan, while, concerning side effects and tolerance, Lipidots gave the best results [173]. Indocyanine green (ICG) and its derivates absorb strongly NIR light and can be used for both photothermal conversion and photodynamic therapy by producing singlet oxygen. Nguyen and co-authors incorporated docetaxel and ICG in low-temperature sensitive liposomal formulations, with an average size of 130 nm, and found that NIR-irradiation after treatment resulted in a better tumor regression effect in SCC-7 tumor-bearing mice [174]. When injected intratumorally, the NIR-responsive drug carriers were found to completely ablate the tumor and inhibit its reoccurrence. However, NIR dyes are highly crystalized, which hinders their encapsulation in small liposomal formulations. Moreover, similar to other light-triggered treatments, PDT is characterized by poor bioavailability, high dosage requirements, self-aggregation in aqueous media, and unwanted side effects, due to the presence of some hydrophobic photosensitizing agents. An additional challenge to overcome is that one should know the exact drug target location, so that to achieve this PDT systems to be activated with light illumination to result in therapeutic outcomes.

### 6.3. Magneto-Responsive Liposomes

**Figure 12:**
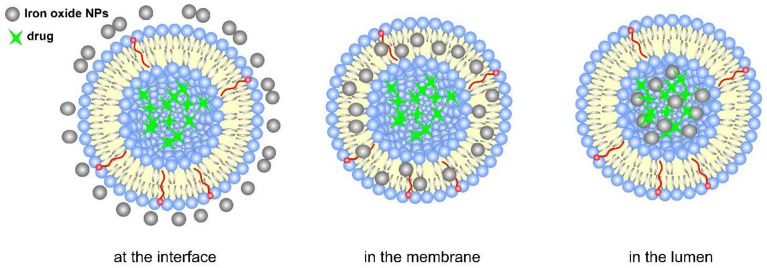
Schematic representation of different types of incorporation of iron oxide NPs in responsive liposomes for targeted therapy with radiofrequency/magnetic fields.

In the recent past, lipid-based nanoparticles that contain magnetic substances were known as magnetic/magnetic liposomes. They had been developed for the diagnosis of miscellaneous diseases. Researchers are now working on the effects of magnetic fields by passing these fields deep into the human body for the treatment of any dreadful disease without harming/damaging healthy tissues. The magnet should provide well-defined field geometry to avoid drug release in non-targeted healthy areas. The biological tissues are highly transparent to magnetic fields, and these magnetic fields are neither transformed nor absorbed by the majority of the biological tissues. Magnetic liposomes (MLs) constitute a versatile delivery system because they exhibit potentiality in various functionality and various combinations of drug delivery, whereas the magnetic nanoparticles are used as drug carriers, which are accumulated in the target tissue with a strong permanent magnetic field. The most used iron oxide nanoparticle is the iron oxide material, which can be injected directly into the tumor by getting exposed to an alternating magnetic field (AMF). It is believed the interaction of the magnetic field with magnetic NPs leads to heat generation that increases the temperature of the lipid bilayer above the phase transition temperature and produces a drug-release effect. The incorporation of NPs may be in the lumen, at the interface of the liposome membrane, and in the membrane (Figure 12), while the latter is thought to be the most efficient [175]. This is because the NPs situated within the membrane can both mechanically (by translational and rotational motions) and thermally (by heat transferred from NPs to the membrane) actuate the release. However, the incorporation of NPs in the membrane could lead to clustering, increased passive release, and micelle formation [176].

Because of their outstanding magnetic behavior, biocompatibility, availability, and easy synthesis, SPIONs NPs (super-paramagnetic Fe_3_O_4_ nanoparticles) are frequently applied to humans [177,178]. For example, synthesized Dox-loaded liposomes of around 200 nm, made of DPPC and iron oxide NPs, are incorporated outside the lipid bilayer. These magnetoliposomes indicated the 100% cellular uptake of MDA-MB-231 and HeLa cancer cell lines, and after applying a remote magnetic field, the cell survival was reduced by 20% [179]. When compared with metallic ions, metallic NPs exhibit properties similar to magnetic targeting, magnetically triggered drug release, and therapeutic hyperthermia. These properties were used by Acharya and Chikan to enhance the functionality of drug-loaded liposomes made of DPPC/DSPC/Cholesterol-PEG-SH particles covered with gold-coated iron oxide NPs. The drug-release efficiency of these formulations under exposure to pulsed magnetic fields indicated that up to 20% of the drug can be released in a short time, compared to bare gold-covered or only iron oxide-coated liposome conjugates [180]. Except for SPIONs and gold NPs, Dai et al. proposed hyperthermia-triggered local drug release by encapsulating responsive magnetic ammonium bicarbonate with Dox in liposomes with a particle size of about 210 nm. When subjected to a permanent magnetic field, the intercellular accumulation of Dox increased, as compared to non-magnetic ammonium bicarbonate-loaded liposomes [181]. The limitation of the approaches applying a permanent magnetic field is rapid heat dissipation in the surrounding tissues and potential heat-induced injury of healthy cells, as well as the need for a high concentration of magnetic NPs.

A potential of controlled drug release is the magnetic liposomes subjected to a low frequency “non-heating” alternating current magnetic field that involves the generation of mechanical forces by single domain magnetic NPs that undergo oscillating movements [182]. The advantage of magneto-mechanical actuation is the relative safety of low-frequency AMF to the human body. For that reason, researchers are trying an attempt to use the AMF to induce drug release through superparamagnetic iron oxide nanoparticles by generating heat via the Neel (internal magnetic realignment) and Brownian (rigid body rotation) relaxation principles, and the energy required to convert them is typically about 100 kHz [183]. It was discovered that, depending on the location and charge of the magnetic NPs, both magnetic heating and oscillation effects can differ. Magnetic heating dominates the release of DOX when negatively charged magnetic NPs are located inside the nanocarrier, while both magnetic heating and oscillation effects are important for the release of the drug when the positively charged magnetic NPs are located on the surface. However, both magnetic responsive Dox-loaded nanocarriers have obvious cytotoxicity against HeLa cells under external AMF [184].

In recent days radiofrequency thermal therapy (RTT) has drawn widespread attention and is considered to be minimally invasive, controllable, and highly efficient in cancer diagnosis [185]. Pan et al. co-encapsulated iron oxide NPs and Dox drugs inside liposomes composed of zwitterionic phosphatidylcholine, anionic phosphatidylglycerol, and cholesterol lipids and coated them with poly-L-lysine enriched shell with gold anions. In A549 human lung cancer cells, the formulation retained its Dox cargo and remained in the cytosol, while, after radiofrequency (RF) or NIR, it triggered the nanostructures released Dox, which entered the cell nucleus. Compared to a single RF or NIR treatment, the combined Dox and RF or Dox and NIR displayed higher therapeutic effects on cancer cells [186].

Liposomes are also able to pass the blood-brain barrier (BBB) that protects nerve cells from many harmful agents and limits the transport of different xenobiotics, as well as anticancer drugs. Drug-loaded liposomes facilitate BBB penetration and constitute an efficient drug transport tool for glioblastoma multiforme. By applying AMF to thermosensitive magnetoliposomes and reaching a temperature of 43 °C for a few minutes, a strong anti-glioma effect associated with complete remission was found, due to a sustainable Dox release [187].

A combination of PDT and hyperthermia methods in ultramagnetic liposomes was also found to enhance therapeutic efficiency. In a study by Di Corato and co-authors [188], liposomes highly loaded with magnetic NPs in the hydrophilic core and containing Foscan photosensitizer were used for cancer therapy. The laser activation of the liposome carriers triggered a change in the surrounding oxygen into a reactive form that, together with magnetic hyperthermia, caused the activation of apoptotic pathways, leading to complete cancer cell death and tumor regression in vivo.

Although the incorporation of magnetic NPs within the membrane demonstrates clear biological benefits related to controlled drug release; still, challenges, such as easy clustering, micelles formation, and increased passive release, are present. AMFs, such as electromagnetic waves, are limited by diffraction that prevents the focusing of alternating fields to resolutions of better than 1 m [189] and encourage nanocarrier accumulation at superficial tissue sites [190], which can diminish the advantages of using magnetism as a drug-release strategy. Moreover, magnetoliposomes require additional tumor-cell targeting ability that will lessen the severe side effects on healthy tissue.

### 6.4. Electrical Energy-Responsive Liposomes

Applying a weak electric field to a cancerous tissue can result in programmed drug delivery, due to disruption of the vesicle structure, redox reaction, or heat production. Electro-responsive materials can be used for protein, DNA, or drag release from conductive polymer or hydrogel materials that are sensitive to low voltage causing electro-responsiveness into dopant ion carriers for producing oxidation or reduction reactions [191]. Such a stimulus can promote the swelling or disruption of nanostructures. Electric fields influence the plasma membrane polarity and intercellular communication to control cell migration, growth, and differentiation [192].

Recently, giant unilamellar lipid vesicles were evaluated for stimulus-dependent response by using a series of electric field pulses with micro- and millisecond duration and were found to be successful for electrofusion, electrodeformation, and electroporation of the membranes [193]. The authors concluded that the formation of electrically-induced pores was in agreement with those reported for mechanical-induced ones. However, electrical therapy is a biophysical chronic treatment with limited effectiveness [194] because irreversible electroporation implies the creation of permanent and lethal pores in the cell membranes, causing cell death of cancer and healthy cells. A more powerful approach could be the use of electric pulses that do not disrupt the cells, but induce an electro-permeabilization process that is based on the creation of transient pores. This technology platform for enhancing the transport of drugs, genetic materials, and other molecules is becoming popular in the areas of medicine, food processing, and environmental applications [195]. In such a way, liposome electroporation can permit drug release in the extracellular medium close to the tumor cells and simultaneous and reversible electro-permeabilization of both cell and liposome membranes for drug exchange. Reversal electroporation can also facilitate liposome accumulation on the tumor site by changing the vascular permeability, thus enhancing EPR effects [196].

To deliver multiple “physicochemically incompatible” chemotherapeutics to oral cancer, Sonaje et al. constructed charged deformable liposomes, termed “iontosomes”, that were able to overcome the buccal mucosal barrier via a combination of electrical potential gradient [197]. The liposomes comprised 1,2-dioleoyl-3-trimethylammonium-propane (DOTAP) and Lipoid-S75 and co-encapsulated two chemotherapeutic drugs—docetaxel and cisplatin. Because of their electro-responsive shape-deformable properties, the formulations were able to penetrate the mucosa through intercellular spaces, while the penetration depth was controlled by varying the duration of the current application.

Compared to other types of external stimuli, due to the precise control of drug release with the magnitude of current and duration of electric pulse, the activity of the electrical energy-responsive liposomes can be well-adjusted. Additionally, no complex instrumentation is needed. However, still, a great challenge of this strategy is obtaining electroporation conditions that trigger release from liposomes, without permanently damaging normal cells.

### 6.5. External Heat-Responsive Liposomes

When the tissue temperature exceeds the normal body temperature (37 °C), hyperthermia is observed. Unfortunately, when relying on minor temperature differences in vivo for triggering payload release, an undesired compromise with the high passive release is needed [198]. In contrast to pH or enzyme-triggered clues, the temperature has the advantage of being able to control by external means. Mild hyperthermia exceeds the physiological temperature by a few degrees, while at the limit of 41–42 °C cells, especially cancer cells, are not able to maintain their normal function [199]. Mild hyperthermia, as adjunctive therapy with radiation and chemotherapy, has long been administered [200].

**Figure 13:**
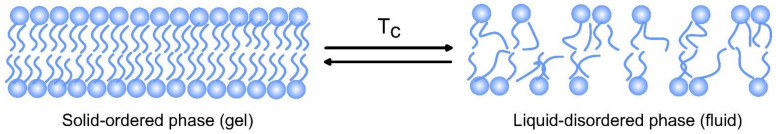
A schematic presentation of “gel” to “liquid” transition of thermoresponsive liposome formulations occurring during mild hyperthermia and controlling drug release.

Temperature-sensitive liposomes (TSLs) are nanoparticles that rapidly (in a few seconds) release the contained drug at hyperthermic temperatures, typically above ~40 °C, and combine with various heating modalities, such as thermal ablation/radiofrequency ablation (RF), to study the changes in drug uptake [201]. TSLs are found to undergo a sharp change in the properties of lipids in the bilayer or other components of the vehicles with temperature. Together with hyperthermia, the TSL can accumulate in tumors or exert direct cytotoxicity to cancer cells, while modulating the tumor vascular permeability and cell susceptibility to the released drug in the area exposed to heat. TSLs, in combination with hyperthermia, can increase therapeutic effectiveness by promoting drug release from the temperature-sensitive formulations into the tumor vasculature and interstitium [202]. TSLs undergo a “transition” upon a slight temperature increase (3–5 °C) from “gel” to a “liquid” phase, where the mobility of the head groups gradually rises, and the orientation of the hydrocarbon chains turns from *trans* to gauche configuration (Figure 13). The disordered interfaces within the solid lipid domains make the membrane highly permeable and facilitate drug release. The thermo-responsive liposome formulations can be traditional, polymer-modified, or lysolipid-containing TSLs [139].

Traditional thermosensitive liposomes were studied in 1978, introducing liposomes that released neomycin and inhibited bacteria protein synthesis in vitro at specific temperatures [203]. These were the first of their kind, now known as traditional TSLs, further developed over the next few decades, and are comprised of lipid membranes that undergo phase transitions in response to heating [204]. Recently, traditional TSLs are comprised of dipalmitoylphosphatidylcholine (DPPC) and distearoylphosphatidylcholine (DSPC), with transitional temperatures between 42–44 °C or higher than the normal body temperature. Although indicating increased drug release, these formulations demonstrated a small amount and low rate of drug release [107]. For that reason, Li et al. constructed vincristine and Dox-loaded TSLs, in which the co-encapsulation of drugs enhanced the biodistribution and pharmacokinetic profile of the DDS to MCF-7 tumor-bearing mice [205]. Hyperthermia increased the liposome accumulation within the tumors, while the accumulation doubled with the rise of temperature from 39 to 42 °C. The observed results proved the synergetic reaction of the EPR effect on tumors and the hyperthermia phenomenon.

The lysolipid-containing TSLs encouraged rapid drug release by decreasing the phase transition temperature. Al-Ahmady et al. investigated Dox-loaded traditional and lysolipid-containing TSLs with protein corona in vivo and ex vivo at 42 °C [206]. In contrast to traditional liposomes, lysolipid-containing TSLs showed ultra-fast and complete Dox release under different tested conditions, indicating the structural composition, corona formations, and protein content of the drug-releasing environment influenced the drug release profile.

The most effective method for heat-sensitizing liposomes is the incorporation of naturally occurring or synthetic polymers in the liposome membrane. The modifying polymers usually have a lower critical solution temperature (LCST), and hydrogen bonding forces between water molecules and polymer chains are sufficient to solubilize the polymer. The phase transition resulted in membrane disruption and the promotion of drug release near the polymer. Such a polymer is poly(N-isopropylacrylamide) (pNIPAM), with an LCST at about 32 °C, which is near body temperature [207]. Another type of thermosensitive polymer is poloxamers which contain the main hydrophobic block of poly(propylene oxide) between two hydrophilic poly(ethylene oxide) chains. Below the critical micelle temperature (CMT) of the poloxamer molecules, the latter do not associate with the lipid bilayer of poloxamer-containing liposomes, while above the CMT, their partition into the lipid bilayer causes membrane disruption and drug release [108]. By adding poloxamer 188 to conventional TSLs, the encapsulating efficiency of the drug increased to 90%, while the drug release was shortened to 10 min at 42 °C, increasing the antitumor activity in nude mice [208]. Except for synthetic polymers, thermally responsive biopolymers, such as elastin-like polypeptides (ELP), also gained considerable attention. ELP-TS liposome formations have been also used for Dox delivery. The incorporation of ELP into the liposome bilayer by covalent linkage showed more than 95% Dox in less than 10 min at 42 °C, while Dox was retained at 37 °C because of the less rigid lipid bilayer and shorter ELP length of the chain [209].

Multifunctional-targeted TSLs conjugated with specific ligands, such as antibodies, folate, peptides, etc., are also designed. For example, by adding K237 peptide to the surface of paclitaxel-loaded TSLs, higher toxicity against SKOV-3 cells and HUVECs, compared to free drug and bare TS-drug-loaded conjugate, was observed, mainly because of the higher cellular uptake through binding of K237 peptide to receptors on the surface of these cells [210].

The sensitivity of cancer cells to abnormal temperatures makes the usage of TSLs a promising approach. The burst release of drugs within the tumor may require reduced drug dosage and side effects on patients. However, various factors are hindering the clinical translation of these liposome-conjugated drug vehicles, such as selecting materials that are both sensitive enough to small temperature changes around 37 °C and safe. Recent achievements on external stimuli-triggered liposomes are tabulated in Table 4.

## 7. Dual-Sensitive Liposomes

Over the past decade, to make the liposome drug carriers more specific and effective, researchers designed multi-stimuli responsive DDS by combining two or more internal, external, or combined triggers in a single drug-loaded vehicle. Dual sensitive liposomes can offer advantages, such as targeting by more than one physiological signal or receptors, better internalization, the release of a higher amount of drug at target sites, reduced normal tissue toxicity, etc. [90]. By combining different stimuli in one liposomal formulation, both higher specificity and multistage drug delivery can be achieved. Together with the improved pharmacokinetic effects and longer persistence at the target site that provides the liposomes, the applied stimulus may alter the lipid structure and destabilize the particle, thus leading to payload release, though only when necessary. However, the engineered dual- or multi-stimuli-responsive have sophisticated nature and should maintain various biological functions simultaneously, which can sometimes be an obstacle to their effective performance.

The most common format of dual-sensitive liposomes that responds simultaneously to different stimuli is revealed in this section.

### 7.1. pH-Regulated Dual Sensitive Liposomes

pH-responsive liposomes conjugated with other release mechanisms have been intensively investigated. For example, ursolic acid-loaded nanophytoliposomes enwrapped in a poly-L-lysine coat and hyaluronic acid were fabricated by Poudel et al. and proven for pH and enzyme responsiveness. HA provided not only targeted superiority, but also enzyme responsiveness. The internalization in CD44 receptor-expressing cell lines was amplified by the EPR effect, as well as active targeting. In vitro and in vivo findings indicated that the smart targeted and dual-responsive DDS has outstanding safety and antitumor efficacy [218].

New liposomal formulations containing one acid-cleaving group (hexahydrobenzoamide) and an amino acid group, together with a redox-sensitive disulfide bond, were investigated for pH and redox responsiveness, respectively, as an anticancer DDS. The liposomes consisting of synthetic functional lipids exhibited pH-promoted cellular uptake and pH-responsive endo-lysosomal escape because of the protonation of the imidazole group that caused proton influx into the endocytic vesicle, followed by its rupture. The higher GSH content yielded a redox-triggered intracellular release of Dox that caused an enhanced antitumor activity against HepG2 cells, due to internal stimuli [219].

Dual pH–pH responsive Dox-encapsulating liposomes containing responsive acid-sensitive peptide (DVar7) to acidic tumor microenvironments to increase the uptake and responsive acid-sensitive phospholipid (DOPE) for improved and controlled release of Dox in tumor cells was developed by Zhai et al. The therapeutic efficacy of the formulations was found to be positively affected by glucose injection, regulating the acidity of the tumor microenvironment in the breast cancer mouse model [220].

Except for internal stimuli, pH-responsive liposomes were conjugated with external stimuli release mechanisms, such as light [221], temperature [222], or ultrasound [223]. For example, peanut-extracted phospholipids were used for the development of liposomal formulations of the size of 1–2 μm for pH, as well as the thermosensitive delivery and release of camptothecin (CPT). The optimal drug release was at pH 6 and 47 °C, while the CPT release showed remarkable anticancer activity against MCF-7 cells with an IC50 value of 17.99 μg/mL [222]. Chen et al. [224] constructed pH-sensitive, NIR-responsive liposomes coated with pH-sensitive poly (methacryloylsulfadimethaxine) and encapsulated with Cypate, Dox, and NH_4_HCO_3_. The liposome formulation showed enhanced cellular uptake and cytotoxicity at pH 6.5, when stimulated with NIR in a mouse breast (4T1) cancer model. The Dox accumulation at targeted sites was increased, suggesting that these liposomes could enhance antitumor efficacy and reduce the systemic side effects of Dox.

### 7.2. Temperature-Regulated Dual Sensitive Liposomes

**Figure 14:**
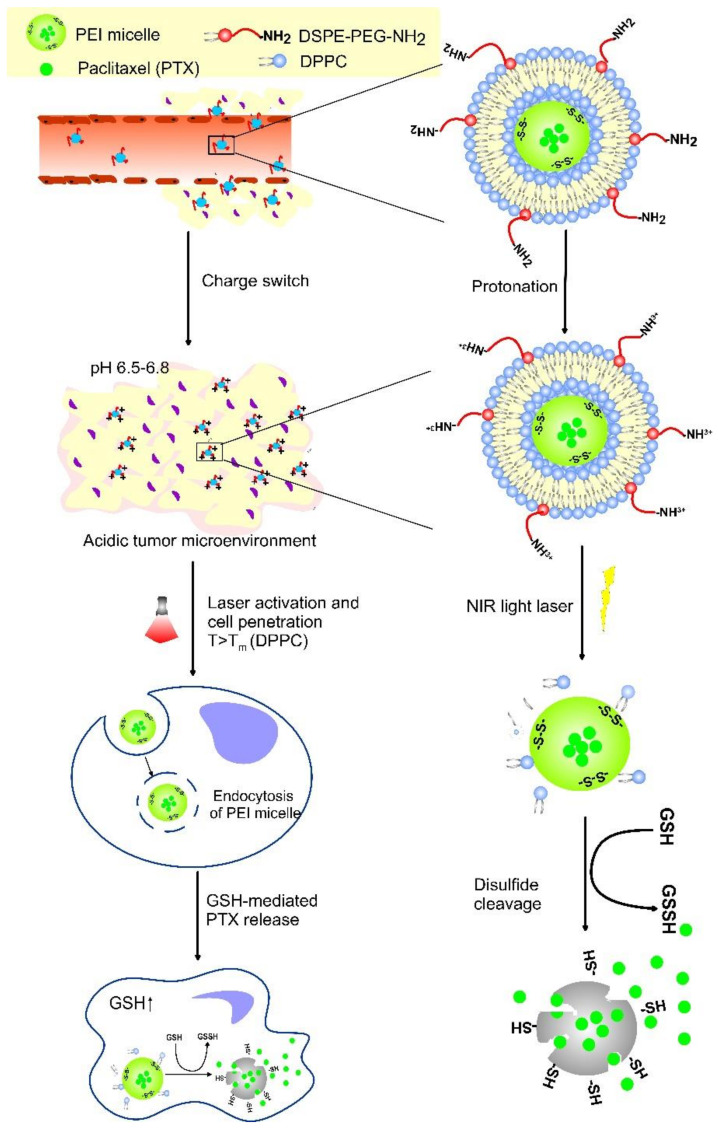
A schematic mechanism of the hierarchical activation strategy of pH, temperature, and GSH-sensitive liposomes for delivery of PTX in cancer tissue.

As mentioned earlier, many studies reported that the dual-stimuli combination, pH and temperature, revealed synergistic effects on cytotoxicity against cancer cells and dramatically decreased the risk of damage to healthy tissue because of the superior therapeutic efficacy of stimuli-activated liposomal cancer therapy [107,225]. A hierarchical activating strategy for enhanced circulation, rapid tumor tropism, facile penetration, and tumor-specific drug release was proposed by Cherukula et al. as a triple-sensitive (temperature, pH, and GSH) liposome platform. The latter consisted of lithocholic acid-conjugated disulfide-linked polyethyleneimine (PEI) micelle, loaded with paclitaxel and subsequently coated with thermosensitive DPPC and DSPE-PEG-NH_2_ lipids (Figure 14). These formulations were laser and pH-responsive, which improved the disposition of therapeutic to the tumor. In the acidic tumor micro milieu (pH 6.5–6.8), the amino groups of DSPE-PEG-NH_2_ lipids, became protonated, which enhanced the liposomal accumulation in cancer tissue. NIR laser irradiation activated the thermosensitive lipids that de-shielded the carrier, and it was disposed to the tumor milieu, thus resulting in enhanced intracellular internalization. After evading the endo-lysosomes, the drug was released through the degradation of disulfide-linked polyethyleneimine micelle mediated by intracellular GSH in the tumor. The multifunctional formulations significantly improved the therapy by eradicating primary tumors completely and suppressing their subsequent lung metastasis [226].

In another study, multifunctional temperature, pH, and NIR light-responsive drug carriers were developed by loading resveratrol (Res) in chitosan-modified liposomes and by coating them with gold nanoshells. The constructed system possessed broad NIR absorbance, stability, high loading capacity, and high photothermal conversion ability. At pH 5, about 57.6% of Res was released, while at pH7.4, this percentage was only 20.5%. Under NIR laser irradiation, the DDS could significantly enhance cellular drug uptake. Compared to a single drug or only photothermal therapy, these formulations with NIR irradiation displayed a higher therapeutic effect against HeLa cells [227].

Promising results were reported by Santos et al. by combining mild hyperthermia of focused ultrasound with lyso-thermosensitive, Dox-loaded liposomes, and in short 30s bursts to 42 °C, measurable amounts of drug were released, thus overcoming the issues that hamper conventional treatments in targets associated with substantial tissue motion [228]. Xing and his group combined light and temperature triggering in the liposomes encapsulating gold NPs and Dox. When irradiated with NIR, the Au NPs were released, while hyperthermia-induced increased membrane permeability of both tumor cells and liposomes facilitated the release and Dox accumulation in cancer tissue. The tumor growth inhibition rate of the multifunctional liposomes was calculated to be around 78% [229]. Shaghasemi et al. proposed magneto-thermally controlled liposome formulations, representing small unilamellar stealth liposomes with SPIONs and the model hydrophilic drug calcein. The release mechanism by local heating of SPIONs in AMF was proposed to obey Neel relaxation. When exposed to AMF, the determining factor for calcein release was the SPION concentration. At a 2 wt% SPION concentration, the first two min pulses released only 28% of the drug, while at 4 wt % SPION, the same duration of pulses released about 48% of the payload [177].

### 7.3. Other Dual-Triggered Liposomes

There are plenty of recent studies demonstrating successful tumor cell identification and the higher cytotoxicity of responsive liposomes to two or more stimuli combined in different ways. For example, Liu et al. developed hypoxia-triggered and hypoxic radiosensitizer liposomes as a Dox carrier against malignant glioma brain tumor that achieved a synergistic chemo-/radiotreatment. The hypoxic radiosensitizer nitroimidazoles were conjugated with lipids by a hydrolyzable ester bond and mixed with DSPE-PEG2000 and cholesterol to form drug-loaded liposomes. The latter were found to have strong radiosensitivity and to promote cargo release in hypoxic conditions. Under hypoxic conditions, the liposomes released 65.8% of their Dox content within 5 h, while no significant drug release was observed under normoxic conditions. These multifunctional liposomes were found to exhibit precise and stealthy pharmacokinetics and efficient passive uptake by the tumor [230]. In another study, Chu H., by using clinically approved NIR fluorescent dye indocyanine green (ICG), constructed magneto-enzymatic sensitive liposomes encapsulating cisplatin. The liposomes contained sphingomyelin that can be hydrolyzed by the stress-related enzyme ASMase excreted in the site of cancer under radiation, hypoxia, or chemical drugs. Additionally, iron particles were added to the lipid bilayer to amplify the activation process by the Brownian motion of the lipids under AMF. The results confirmed that the liposomal cisplatin release increased with increased radiation doses and increased ASMase activities. Oral squamous carcinoma (SCC9) cells were less sensitive than hypopharyngeal squamous carcinoma (UDSCC2) cells under high doses of radiation. However, a temporary increase in the ASMase activity of extracellular induced by cisplatin released by radiation was not observed in vivo [231]

Recently, GSH-sensitive and ultrasound-triggered Pt(IV) prodrug-loaded phase-transitional NPs, composed of a perfluohexene (PFH) liquid core, a hybrid lipid-polymer shell with PLGA_12k_-PEG_2k_ and DSPE-PEG_1k_-Pt(IV), and an active targeting ligand—cRGD peptide (cyclic Arg-Gly-Asp)—were developed by Huang et al. A platinium(IV)-based anticancer drug (Pt(IV) NP-cRGD) exhibited excellent echo-persistence under an ultrasound field. GSH-sensitive and ultrasound-triggered DDS increased the therapeutic effect and decreased the toxicity of chemotherapy for ovarian cancer. The formulation by the consumed GSH and enhanced ROS levels further caused mitochondria-mediated apoptosis [232]. In another study, dual NIR-light and redox-responsive liposomes with hydrophobically modified photosensitizer (ICG-ODA) and encapsulating Dox were further modified with Her2 antibodies to endow targeting ability toward Her2 receptor-positive tumor cells. Under NIR-light, the formulation produced ROS by ICG, due to the PDT effect. ROS oxidized vinyl ether bonds of special lipid molecules, leading to structural disorder and Dox release. Thanks to the enhanced accumulation and specific Dox release under laser irradiation, an extraordinary tumor growth inhibition effect, based on the MCF7 and SKOV3 tumor models, was observed, together with low systematic toxicity of the therapeutic process [233]. Other recent studies, concerning some achievements of the dual-sensitive liposomal formulation, are revealed in Table 5.

### 7.4. On-Off Switch Regulated Liposomes

Since liposomes can lose their therapeutic drug by leaking before reaching the cellular target, the concept of chemical switches responding to environmental conditions has also been developed. The switch can be interconverted between two state-defined environmental conditions (pH, redox, UV, temperature, etc.) present inside or outside the liposome. When activated, the clue-responsive trigger releases the original drug molecule. Liposomes with “On” and “Off” responsive glucosidase (triggered by reaction with lysosomal beta-glucuronidase) switches [245] and thermosensitive leucine zipper lipopeptide anchored in the liposomal bilayer [246] have been constructed. Using the insertion of a copolymer consisting of an azobenzene group linked with PDPA, reversible get-in and get-out blocks in the liposome bilayer were obtained that could ultimately realize complete drug release under UV-irradiation and pH stimuli [247]. The combination of such switches could be also useful for precise targeting of the liposomal formulations, with tuneable control over on-demand drug release.

### 7.5. Gel-Type Regulated Liposomes

Hydrogel can be considered a promising material for drug delivery systems because of its biostability, biodegradability, and ability to adapt its mechanical properties and degree of swelling for a specific application. When using stimuli-responsive hydrogels as DDS, the diffusion pattern of the drug, through the hydrogel matrix, should be regulated by the composition, crosslink density, mesh size, and interaction between polymer and drug. Such hydrogels can incorporate liposomes to surpass uncontrolled release and liposomal instability or can be located in the liposome core/inner aqueous cavity.

Different responsive hydrogels have recently been explored in various drug delivery systems. Thermosensitive hydrogels used as drug carriers can not only undergo phase transition (swell/de-swell) at ambient temperature but also endow the DDS with high local drug penetration, improved drug bioavailability, and desirable temporal and spatial control [248]. Similarly, photo-sensitive hydrogels with UV or Vis light activation demonstrated tunable properties and excellent biocompatibility [249]. However, because the latter, hydrogels require the activation of the photosensitive chemical group by photonic energy, the limited depth of penetration to the tissue is an issue in their biomedical application. The presence of magnetic NPs in hydrogels allows for the magnetic targeting of a DDS. Mart et al. invented a DDS in which alginate gel was successfully used for the incorporation of magnetic vesicles. The system not only responded to AMF and enabled the drug-controlled release, but also was sensitive to host enzymes/glycolipids [250]. Ulllrich at al. [251] developed alginate magnetic lipogels loaded with laccase in the hydrogel matrix and a substrate 2,2′-azinobis(3-ethylbenzthiazoline-6-sulfonate) in the liposome. The system was able to perform a catalytic function, due to the immobilized enzymes, as well as present storage function because the liposomes acted as a reservoir of the molecule cargo, as well as communication through a translation of the radiofrequency signal into a biochemical reaction because of the higher permeability of the liposomes membranes. Due to this higher permeability of liposomes and the difficulties in transferring drugs into the abdominal cavity, Sugiyama et al. tried to formulate temperature-sensitive paclitaxel (PTX)-loaded gelation liposomes for treating peritoneal dissemination, focusing on enhancing retention time in the abdominal cavity. The hydrogel-contained cellulose and citric acid and became gelated at body temperature. Liposome PTX suspended in temperature-sensitive gel had a delayed leak of the drug. In an animal model with mouse ovarian sarcoma M5076 cells transplanted intraperitoneally, the liposomal formulation decreased the number of cells to approximately 58% of the control level (free PTX) because of the sustained leak and increased retention period in the abdominal cavity. It was also concluded that the liposomal PTX was more cytotoxic at lower concentrations than hydrogel-embedded PTX because liposomes directly approached the tumor cells leaving the gel [252].

DDSs with multiple stimuli-responsive abilities allow for identifying the tumor tissue more accurately among healthy cells. For example, only tissue microenvironments with acidic pH, higher expression of tumor-specific enzymes, and reductive environment can be recognized as target areas that will improve the specificity of anti-tumor therapy.

## 8. Conclusions and Future Prospective

The rapidly developing field of stimuli-responsive liposomes has already shown its effectiveness in drug delivery to cancerous tissues or organs. The biological difference in healthy and diseased cells gives the potential to progress in the tumor disease field, thus achieving better biodistribution and enhanced targetability of drugs. Simultaneously, liposomes appear to be one of the healthiest, safe, and most effective nanoformulations developed so far. Composed of naturally occurring lipids, these drug carriers can be easily metabolized in the body, so they can be regarded as biodegradable and biocompatible drug vehicles.

The therapeutic benefits of liposome-encapsulated anti-tumor drugs include reduced toxicity and improved treatment efficacy. However, only a liposome drug-vehicle targeting itself by specific ligands toward tumor cells is not always sufficient for achieving successful therapy capable of curing cancer. Liposomes allow for easily adjusted targeting modifications and stimuli-responsiveness that will make them successful in personalized therapy focused on each individual or disease. Targeting ligands are often combined with stimuli for better-localized distribution and chemotherapeutic release with minimal systemic exposure. To enhance the therapeutic efficacy and overcome certain limitations, polymer-modified liposomes, as well as the conjugation of target-specific ligands that increase drug retention in tumors and improve therapeutic outcomes of liposomal chemotherapy, have been proposed. The progress from single function to multifunctional responsive liposomes has demonstrated huge potential for targeted delivery of therapeutics.

However, some limitations should be addressed shortly, including the safety of the modified components, the change of the compounds in the diseased conditions, their toxicity, and the difference in the efficacy of DDS in both in vitro and in vivo conditions in the presence of stimuli. It is equally important for the liposomes to achieve target-oriented delivery of payloads and then to be safely metabolized or excreted from the organism. Various important properties, such as prolonged circulation in time, serum stability, efficient triggering release, limited absorption, etc., are crucial to optimize. To prevent renal cleaning, enzymatic degradation, physical entrapment, and evading capture by the reticuloendothelial system on the liposomes’ way to the desired target site, their size, surface charge, and functionalization should sustain durable blood circulation. The density of the targeting ligands with PEG should be precisely determined to ensure effective targeting and in vivo potency. Moreover, for multi-drug encapsulating liposomal vehicles, it is crucial to manage the multidrug ratio, depending on the drug yield percentage, in order to maintain the hydrophobicity of the particles and develop synergetic effects [253].

The development of combined multifunctional liposomes with different release mechanisms, such as light-sensitive, magnetic-, temperature, redox-, and ultrasound-responsive could be helpful for particular tumor types in individual therapies. There are a large number of credible studies on successful surface modifications of drug-loaded liposome carriers with on-demand release. Liposomes, thus, being one of the best DDSs, have to be extensively translated into clinical practice. However, there are also studies reporting a gap between the targeted surface modifications and clinical trials in human models. Animal models for treatment cannot be extrapolated to humans. Some studies suggest that this is because of the difference in tumor pathophysiology, which differs in the human and animal models used for research [254]. However, the individual tumor pathophysiology has to be well-examined by reliable and fast techniques because, nowadays, precision medicine oncology is based on biomarkers (tumor-specific protein overexpression) that cannot only be used for selective targeted therapy, but can be tailored to each patient in line with his/her tumor molecular profile [255]. It is also important for the patients to obtain their personalized dosing treatment in dependence on the targeted liposomal penetration in the tumor tissue [256]. To detect penetration, the non-invasive imaging techniques by utilizing radiolabeled liposomes or loaded with dyes, MRI contrast agents, etc., may provide useful information for predicting and calculating the drug therapeutic delivery.

Additionally, large-scale production requires the selection of appropriate solvent and fabrication methods, estimation of cost, etc. The sensitivity, specificity of identifying tumor lesions, and the safety and toxicity of the final formulations should be assessed before scaling up [257]. Moreover, despite the advantage of the high purity of the chemical derivates of phospholipids, their high cost remains a challenge. For that reason, naturally occurring phospholipids can be purified. Therefore, the clinical translation of these liposome formulations still needs significant investigation.

Because liposomes can deliver thousands of payload molecules by attaching a few ligands on their surface, they should be pursued and diversified. Although many issues remain to the realization regarding full liposomal potential, growing interest in the development of stimulus-responsive DDS for cancer treatment will result in substantial improvement in the life quality of patients.

## Figures and Tables

**Table 1:** Summary of the FDA/EMA-approved liposomal products.

Drug/Active Agent	Product (Year Approved)	Administration Route	Polymer Structure (Diameter)	Drug Encapsulation Amount	Indication	Ref.
Doxorubicin	Doxil^®^ Caelyx (1995, US) (1996, EU)	Intravenous	PEGylated high cholesterol liposomes (SUVs) of HSPC, MPEG-DSPE, Chol (80–100 nm)	Lipid to drug ratio = 8:1 (more than 90% Dox encapsulation)	Ovarian cancer, AIDS-related Kaposi’s sarcoma, myeloid melanoma	[29,75]
Daunorubicin	DaunoXome^®^ (1996, US)	Intravenous	Non-modified high cholesterol liposomes containing DSPC, Chol (45–80 nm)	Lipid to drug weight ratio 18.7:1	Blood cancer, Kaposi’s sarcoma	[29,76]
Cytarabine/ Ara-C	DepoCyt^®^ Depocyte (1999, US) (2001, EU)	Intrathecal injection (Spinal)	Non-modified multivesicular particles consisting of DOPC, DPPG, Chol, Triolein (3–30 μm)	-	Neoplastic meningitis and lymphomatous meningitis	[29,77]
Doxorubicin	Myocet^®^ (2000 US) (2000, EU)	Intravenous	Non-modified liposomes constructed of EPC and Chol (90–250 nm)	Drug to lipid ration = 0.27	Combined therapy with cyclophosphamide in metastatic breast cancer	[29,78]
Mifamurtide-PE	Mepact^®^ (2004, US) (2009, EU)	Intravenous	Non-modified multilamellar vesicles of POPC, OOPS (less than 100 nm)	-	Non-metastatic osteosarcoma	[29,79]
Vincristine sulfate	Marqibo^®^ (2012, US)	Intravenous	Non-modified optisomes containing SM and Chol (approximately 100–150 nm)	95%	Acute lymphoblastic leukemia	[29,64]
Doxorubicin	Lipo-Dox (2012, US)	Intravenous	PEGylated liposomes	-	Breast and ovarian cancer, Kaposi’s sarcoma	[80]
Paclitaxel	Lipusu (2013, US)	Intravenous	Non-modified liposomes (400 nm)	99%	Gastric, ovarian, and lung cancer	[81]
Irinotecan hydrochloride trihydrate	Onivyde^TM^ (2015, US) (2016, EU)	Intravenous	PEGylated unilamellar liposomes containing DSPC, MPEG2000-DSPE, and Chol (110 nm)	90%	Combined therapy with leucovorin and fluorouracil in metastatic adenocarcinoma of the pancreas	[29,82]
Daunorubicin and cytarabine (1:5)	Vyxeos^TM^ (CPX- 351) (2017, US) (2018, EU)	Intravenous	Non-modified low cholesterol bilamellar liposomes of DSPC, DSPG, Chol (110 nm)	-	Acute myeloid leukemia (AML)	[83]

Abbreviations: Chol—cholesterol; DSPC—distearoylphosphatidylcholine; DSPG—distearoylphosphatidylglycerol; MPEG2000-DSPE—N-(carbonyl-methoxypolyethyleneglycol-2000)-distearoylphosphatidylethanolamine, SM—sphingomyelin; POPC—palmitoyl-2-oleoyl-sn-glycero-3-phosphocholine; EPC—egg phosphatidylcholine; DPPG—dipalmitoylphosphatidylglycerol; DOPC—dioleoylphosphatidylcholine; HSPC—fully hydrogenated soy phosphatidylcholine; MPEG-DSPE—N-(carbonyl-methoxypolyethyleneglycol)-distearoylphosphatidylethanolamine.

**Table 2:** Overview of recently registered patents on targeted liposome formulations.

Company	Year	Drug	Active Targeting/Stimuli	Indication	Patent №
Beijing Greatsun Bio Pharm Tech Co., Ltd.	2020	Dox	c(RGD-ACP-K)	Long-circulating drug-loaded liposomes targeting tumors with high expression of integrins α_V_β_3_	EP3632413A1
Celsion Corp.	2019	Dox	Temperature	Increased drug circulation time; deceased drug uptake by the reticuloendothelial system	US10251901B2
Institute of biophysics, Chinese Academy of Sciences	2019	Dox	Heavy chain human ferritin	Solid tumors and hematological cancers	US10195155B2
Oncology venture ApS (Denmark)	2019	Cisplatin	Phospholipase A2	Cancer treatment	EP3342879B1
Temple University	2019	Vinblastine, bevacizumab, and verapamil	Her-2/neu	Breast cancer	US10188728B2
University of Michigan	2019	Mitoxantrone	Hyaluronic acid (HA)	Cancer treatment	US10307491B2
Merrimack Pharmaceuticals Inc (US)	2018	Taxane (taxane prodrug) and a second agent (T-cell regulatory)	Anti-Epha2	Ephirin type-A receptor-expressing tumors	
LEAF Holding Group LLC	2017	Gemcitabine	Hypoxia	Pancreas, lung, ovarian, bladder, breast cancer	US20170319482
Amoabediny Ghasem Tehran, University of	2016	Co-encapsulated two herbal drugs (silibinin and glycyrrhizic acid)	Monoclonal antibody (anti-CD147, anti-CD20, anti-HER2, anti-VEGF-A, etc.)	Cancer treatment	US20160228362
American University of Sharjah	2017	Chematoterapeutic drug (calcein, Dox, vincristine, paclitaxel, etc.)	Ultrasound and trastuzumab (monoclonal antibody)	Cancer treatment	US10864161B2

**Table 3:** Recent studies focusing on internal stimuli-activated liposomal formulations.

Intern. Stimuli	Formulation	Anticancer Drug	Cell Line/Animal Model Tested	Main Finding	Ref.
pH	Polypeptide DVar7 with DSPE-PEG_2000_-MAL	Dox	Breast cancer (MDA-MB-435S) cells; Tumor-bearing female nude mice	- High encapsulating activity (98%); - Good stability in vitro; - Acid-sensitive controlled drug release - pH-sensitive liposome has the best tumor suppression.	[129]
	Liposomes of hydrogenated soy phosphatidylcholine (HSPC) and HA grafted with functional 3-diethylaminopropyl (DEAP) groups and	Docetaxel (DTX)	Human colon carcinoma (HCT-116) cells	- Liposomes with a molar ratio DEAP/HA 0.4 allowed efficient drug release at pH 6.5; - Liposomes were entrapped with cells overexpressing the CD44 receptor; - Significant increase in tumor cell death.	[130]
	1,5-dihexadecyl N,N-diglutamyl-lysyl-L-glutamate (GGLG) liposomes conjugated with Fab′-fragment of ErbB2 antibody to the terminus of PEG	Dox	Breast cancer (HCC1945) and MDA-MB-468 cells; Female bulb/c nude mice	- The cell association of Fab′-GGLG increased 10-fold in comparison to bare GGLG liposomes; - Enhanced Dox intercellular delivery and cytotoxicity in cell lines; - Tumor growth inhibition in ErbB2 overexpressing breast cancer-bearing mouse.	[131]
	RGD co-modified with [D]-H_6_L_9_ liposomes	PTX	Colon carcinoma (C26) and breast cancer (MCF-7) cells; Bulb/c mice	- Under pH6.3 the DDS was taken by C26 and C26 tumor spheroids with significant efficacy compared with other groups; - RGD could decrease cellular uptake of the liposome while [D]-H_6_L_9_ could increase it; - Increased cellular toxicity against C26 cells compared to liposomes with only [D]-H_6_L_9_ or RGD.	[132]
Redox	Paclitaxel-SS-lysophosphatidylcholine prodrug containing EPC/Chol/mPEG_2000_-DSPE	Paclitaxel	Breast (MCF-7) and lung (A549) cancer cells	- Liposomes dissociated rapidly in the reduction medium; - The formulations exhibited GSH-mediated anti-proliferative; - Activity as opposed to non-responsive counterparts.	[133]
	Liposomes of disulfide phosphatidylcholine, 1,2-distearoyl-sn-glycerol-3phosphoethanolamine-PEG_2000_ and cholesterol	Paclitaxel (PTX)	Breast (MCF-7) and lung (A549) cancer cells; Balb/c mice	- Improved efficiency, biodistribution, and safety compared to the drug and non-sensitive PTX liposomes; - Improved antitumor activity.	[134]
	HA	Dox	Osteosarcoma (MG63) and normal liver (LO2) cells; Bulb/c nude mice	- Adding 10 mM GSH triggered burst release of Dox of over 60%; - More pronounced cytotoxicity to MG63 than to normal LO2; - Significant inhibition of tumor growth compared with free Dox or other liposomes;	[135]
	Estrogen-functionalized cationic liposomes linked with chitooligosaccharides (COS)	Dox	Osteosarcoma (MG63) cells and liver (LO2) cells; Male balb/c nude mice	- The formulations were GSH-sensitive and stable in physiological conditions; - Higher cytotoxicity to MG63 than to normal LO2; - The multifunctional liposomes selectively accumulated in MG63 xenografts vs. the organs; - Strong inhibition of tumor growth and enhanced animal survival rate.	[136]
Enzyme	Β-cyclodextrin modified MMP-2 responsive liposomes	Antifibrosis pirfenidone (PFD) and chemotherapeutic drug gemcitabine (GEM) against pancreatic cancer	PSCs/Panc-1 mice xenograft	- About 75% of the drug was released after 2 days of the MMP-2 treatment; - Increased drug perfusion without any overt side effects.	[137]
	sPLA2 sensitive liposomes	Oxaliplatin	Human colon cancer (HT-29 and Colo205) cell lines; Mice bearing FaDu tumors	- Cell growth inhibition by 50% in both cell lines; - In vivo systemic toxicity; - Multiple high dosages showed petechial cutaneous hemorrhages and multifocal hepato-necrotic lesions because of premature activation in the skin and liver, respectively.	[138]

**Table 4:** Recent studies focusing on external stimuli triggered liposomes.

External Stimuli	Formulation	Load	Cell Line/Animal Model Tested	Main Finding	Ref.
Temperature	Thermosensitive liposomes	Dox	Nude mice carrying subcutaneous Lewis lung carcinoma	- Hypothermia duration predicted the tumor drug uptake with drug concentrations of about 4.2 μg/g (no HT), 7.1 μg/g (15 min HT), 14.1 μg/g (30 min HT), and 21.4 μg/g (60 min HT).	[211]
Light	NIR-responsive bubble-generating thermosensitive liposome	Dox, Cyparate, NH_4_HCO_3_	MCF-7 cell line; Female Bulb/c nude mice	- NIR-induced NH_4_HCO_3_ decomposition and formation of CO_2_ bubbles; - The formulations with irradiation damaged the cells more severely than the groups without irradiation; - In vivo results showed dramatically increased Dox in the tumor, inhibited tumor growth, and reduced systemic effects of Dox.	[212]
Magnetic field	PEGylated liposomes	Dox and magnetic NPS	L-929 fibroblasts and HeLa cells	- No cytotoxic effects against fibroblasts; - The cytotoxicity of the released Dox was a function of Dox concentration.	[213]
Magnetic field	Thermosensitive magnetic liposomes with an aqueous core and surface-conjugated Cetuximab (CET)	Camtosar and citric acid-coated Fe_3_O_4_ NPs	Human primary glioblastoma cells (U87); Mice orthotopic xenograft brain tumor model	- At 43 °C, the liposomes undergo burst release of the drug; - CET-mediated tumor endocytosis, high biocompatibility, enhanced tumor cytotoxicity, and no hemolysis in vitro; - the therapeutic efficacy was confirmed in the mouse model.	[214]
Ultrasound (US)	Surface functionalized with Indium-111 tagged epidermal growth factor liposomes	Dox	Human breast cancer (MDA-MB-468, MCF-7) cell lines; Mice bearing subcutaneous MDA-MB-468 xenografts	- Selective uptake in MDA-MB-468 cells compared to MCF-7; - Dox was released in the intercellular space and shuttled to the nucleus; - Dox and Indium 111 had an additional cytotoxic effect on MDA-MB-468; - US application in vivo increased tumor uptake by 66% despite poor vascularization of MDA-MB-468 xenografts.	[215]
US	Stealth liposomes conjugated with Human serum albumin (HAS)	Calcein	Breast cancer (MDA-MB-321 and MCF-7) cell line	- Calcein uptake was enhanced by both cell lines after sonification	[216]
US	PEGylated liposomes conjugated to HA	Calcein	Breast cancer (MDA-MB-321) and NIH-3T3, an embryotic mouse fibroblast	- HA increases the cellular uptake in MDA-MB-321 for its CD44 receptor overexpression; - US enhanced calcein uptake by MDA-MB-321 following sonification.	[217]

**Table 5:** Recent studies focusing on dual-sensitive liposomal formulation.

External Stimuli	Formulation	Load	Cell Line/Animal Model Tested	Main Finding	Ref.
pH-temperature	DPPC and pH-sensitive octylamine grafted poly aspartic acid (PASP-g-C8)	Cytarabine (CYT)	Human hepatoma (HepG2) cells.	- Significant pH-temperature response and prolonged release compared to control liposomes; - The formulations had 30% higher cell apoptotic effects than the free drug;	[234]
pH-temperature	Poly NIPAAm-co-PAA (poly NPA) in liposome formulation	Dox and mitomycin C	Normal fibroblast (NIH3T3) cell line and breast cancer (MCF-7) cell line.	- The maximum leakage at 37 °C and pH 7.4 was 15% confirming the stability of the multifunctional conjugates. - At temperatures between 40 and 45 °C the drug release rose to about 71%; - At pH 5.5 and temperature between 40 and 45 °C the liposomal formulations achieved a release value of 98%; - No substantial cytotoxicity was found for the normal cells at a concentration of 40 μg mL^−1^.	[225]
NIR light and temperature	2-(4-aminophenyl) benzothiazole (CJM126) coupled with cholesterol in liposomes containing folate-PEG_2000_-DSPE	Cisplatin and indocyanine green (ICG)	Breast cancer (MDA-MB-231) cells.	- Under NIR irradiation liposomes showed lower cell viability (3.05%) compared to free drugs or treatment without NIR.	[235]
pH, temperature, NIR light	Gold nanoshells coated liposomes mediated by chitosan	Oleanolic acid	Human osteosarcoma (143B) cells.	- The release rate was higher (~53%) at pH5.5 than at 7.4 (42%); - The drug release rate of the NIR group reached about 92% in contrast to the non-NIR group (69%); - The treated cells exhibited a tumor inhibition rate of over 73% without NIR and about 87% with NIR.	[236]
pH and temperature	Crosslinked polyacrylamide copolymers functionalized with cholesterol-modified DNA motifs that undergo gel-to-sol transformations	Calcein and DiIC18(5)	Human hepatoma (HepG2) cells.	- Temperature and enzyme-responsive release from the hydrogel of liposomes.	[237]
pH and temperature	PEG hydrogel-linked temperature-sensitive liposomes and MMPs-peptide crosslinks	Dox	Human aortic adventitial fibroblasts (AoAF) and murine NIH3T3 cells.	- Several hours after exposure to MMP-1 the enzymatic degradation began, then immediate Dox released followed, and then hyperthermic stimulus completed the release for 48 h.	[238]
pH and temperature	Liposomes consisting of 1,2-dipalmitoyl-sn-glycerol-3-(cytidine diphosphate), DPPC, and cholesterol anchored with NH_2_-PEGylated gold NPs	Dox	Breast cancer (MDA-MB-231) cells and SK-OV-3 ovarian cancer cells.	- The nucleolipid presence in the phospholipid structure provided a negative charge and specific recognition of nucleoside fragment - Almost 100% of Dox was released at pH 5.1 and 42 °C compared to about 65% at pH 7.4 and 37 °C; - With the extension of treatment time from 2 to 72 h the liposomal formulation showed higher toxicity;	[239]
pH and magnetic	pH-responsive H_7_K(R_2_)_2_ peptide conjugated liposomes with iron oxide NPs (SPIONs)	PTX	Human breast carcinoma (MDA-MB-231) cells; female Balb/c nude mice.	- IC_50_ value of the formulation was significantly reduced at pH 6.8 (around 7.24 μM), as opposed to that at pH 7.4 (about 32 μM) - The tumor growth inhibition of the formulation was about 90%.	[240]
pH and enzyme	Liposomes with anti-PD-L_1_ peptide and MMPs-responsive moiety	Dox	Mouse melanoma model (B16F10) cells; Female C57BL6 mice.	- The formulation achieved the optimum tumor suppression efficiency (about 78.7%) compared to liposomes containing only Dox (<40%) because of the synergetic contribution from the increase in M6PR expression in tumor cells and the blockade of immune checkpoints.	[241]
NIR light and temperature	Folic acid-modified liposomes encapsulating gold nanorods and drug	Dox and Au NPs	Mouse breast cancer (4T1) and mouse-origin fibroblast (NIH3T3) cells; Balb/c mice.	- NIR pulses for 5 min triggered Dox release while at pH5.5 in 60 min the drug release reached about 46.4%.	[242]
NIR light and magnetic	Coumarin 6-loaded liposomes modified with hyaluronic acid (HA) and embedded citric acid-coated magnetic NPs	DTX	Breast cancer (MCF-7) cells and mouse-origin fibroblast (NIH3T3) cells.	- NIR illumination of the DDS released over 20% more drugs than non-NIR treated; - IC_50_ value of the formulation under irradiation reached about 0.69 μg mL^−1^ while that of the free drug was about 8.93 μg mL^−1^.	[243]
Temperature and magnetic	Thermosensitive (DSPC, DPPC, chol) liposomes modified by MAB1031 antibody to target overexpressed MMPs	Dox and gadolinium-chelate for enhanced MRI	Breast carcinoma (MDA-MB-231) cells.	- After 24 h the drug release from the multifunctional system was about 21.3% at 37 °C; - At 40 °C the Dox released reached 88% for an hour; - The cellular binding of the liposomal formulations was effective.	[244]

## Data Availability

Not applicable.
